# Extracellular and Membrane Protein: Structure, Biological Functions, Diseases, and an Emerging Modality for Drug Discovery

**DOI:** 10.1002/mco2.70667

**Published:** 2026-03-19

**Authors:** Mengqing Zhao, Wenhao Yin, Jianjian Han, Huimin Wang, Zheng Liu, Lilong Liu, Wuxiang Mao

**Affiliations:** ^1^ State Key Laboratory of Biocatalysis and Enzyme Engineering Hubei Province Key Laboratory of Industrial Biotechnology School of Life Sciences Hubei University Wuhan China; ^2^ Department of Urology Tongji Hospital, Tongji Medical College, Huazhong University of Science and Technology Wuhan China

**Keywords:** drug discovery, extracellular proteins, LYTAC, membrane proteins, PROTAC, TPD

## Abstract

Extracellular and membrane proteins serve important roles. They manage cellular communication, structure support, and immune defense. When they malfunction, it cause many diseases like cancer, neurodegeneration, and cardiovascular disorders. Targeted protein degradation (TPD) is a promising therapeutic strategy and aims to remove these faulty proteins. This approach goes beyond traditional drugs, which only block the active site of proteins. The aim of TPD is to entirely remove the targeted proteins in cells. This review began with explaining the structure and functions of extracellular and membrane proteins, highlighting their connection with disease. It then went on to discuss new strategies for their degradation. These emerging strategies include those that take advantage of cell‐surface receptors to target lysosomes, intracellular lysosomal sorting tools, E3 ligases, and nanoparticle‐based systems. A comparison of different TPD tools was also provided. Discussion compared strengths and weaknesses of approaches with small molecules, antibodies, nanobodies, and aptamers. Finally, the review outlined future directions for advanced TPD strategies. Next steps would be the combination of degraders with therapeutic antibodies. Another research interest is the utilization of tissue‐specific receptors from genetic databases. Moreover, the application of TPD to immune and neurodegenerative diseases is also a critical goal for the future.

## Graphical abstract

Targeted protein degradation represents a paradigm shift from traditional protein inhibition to complete protein elimination. This is achieved through three main strategies for degrading extracellular and membrane proteins: recruitment of cell‐surface LTRs, engagement of intracellular proteins, and receptor‐independent mechanisms.

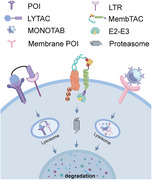

## 1 Introduction

For multicellular systems to function, cells must communicate, collaborate, and be structured [1]. Extracellular and membrane proteins are vital components of multicellular systems. These proteins are considered the primary connection between cells and their surroundings. Membrane proteins embedded in the cell membrane execute critical functions, including signal transduction, nutrient uptake, and cell–cell recognition [2]. Extracellular proteins provide structural support to tissues and function as signaling molecules, including cytokines, growth factors, and antibodies. Together, these proteins establish the sophisticated communication networks that maintain physiological homeostasis [3].

Since they are so important, dysfunction of extracellular and membrane proteins cause many serious human diseases. For example, mutated receptors like EGFR drive cancer growth, while misfolded proteins such as amyloid‐β in Alzheimer's disease form toxic aggregates that harm brain function [4, 5]. Traditional therapeutic approaches have tried to block these proteins with small molecule inhibitors or antibodies. But this approach faces significant limitations, including drug resistance, challenges in targeting “undruggable” proteins, and transient therapeutic effects.

These challenges catalyzed a paradigm shift from protein inhibition to complete protein elimination, which termed TPD. Early TPD approaches, like PROTACs, successfully degraded intracellular targets but could not address proteins located extracellularly or on the cell surface [6]. This limitation is particularly significant because extracellular and membrane proteins constitute approximately 40% of the human proteome. This unmet need has driven the creation and development of new TPD tools. For instance, lysosome‐targeting chimeras (LYTACs) guide cell‐surface receptors to shuttle extracellular and membrane proteins to lysosome for degradation.

This review summarizes recent advances in this rapidly growing field. First, we discuss about the structure and biological functions of extracellular and membrane proteins, explaining how these lead to diseases. Then, we examine and compare diverse TPD strategies for degrading these challenging targets. This included methods that utilize cell‐surface receptors, engage intracellular sorting systems, or employ UPS systems. Finally, we evaluate the various molecular tools employed in these strategies, such as small molecules, aptamers and antibodies, and discussed their respective advantages and limitations.

## 2 Structural Diversity of Membrane and Extracellular Proteins

Membrane and extracellular proteins exhibit distinct structural features that reflect their specialized functions and environments. Membrane proteins reside within the cell's lipid bilayer, and therefore interface with both the hydrophobic membrane interior and the watery areas outside the cell [7]. In contrast, extracellular proteins function in the out space of cells, which contains many enzymes that degrading proteins, necessitating exceptional structural stability. Once secreted, they operate independently, beyond the reach of cellular quality control mechanisms. This section described the structural basis of these proteins for their biological functions and their targeting by the following modalities.

### 2.1 Structures of Membrane Proteins

Membrane proteins make up about 20–30% of most proteomes and represent target for over 60% of modern drugs [8, 9]. These proteins are classified into two categories: integral membrane proteins, which are permanently embedded within lipid bilayer, and peripheral membrane proteins, which associate transiently with membrane surfaces [10]. Integral membrane proteins typically have two parts, containing hydrophobic domains that traverse the lipid bilayer and hydrophilic regions that stick out into the watery spaces outside the cell [11]. Their transmembrane domains commonly adopt α‐helical or β‐barrel conformations, arranged in single‐pass or multipass configurations [12]. Cell membranes act as the final gatekeepers for many substrates and drugs. But studying the structure of membrane proteins has been very slow because it is a challenge to purify them and keep their structure stable [13]. However, this problem has now been solved by new technologies, which has started a new era in membrane protein science. Techniques like cryo‐electron microscopy, X‐ray crystallography, and computer modeling are providing very detailed pictures of these structures [14, 15, 16]. These developments in methodology are giving us new insights into how these molecular machines work. The promising research field include the structural analysis of higher‐order complexes, such as G protein‐coupled receptor (GPCR) arrays, oligomeric transporters, and the synaptic SNARE complex within native lipid environments [17, 18, 19].

### 2.2 Structures of Extracellular Proteins

Extracellular proteins are mostly located in the extracellular matrix (ECM), which is composed of macromolecules that form complicated three‐dimensional networks. Major parts of the ECM are fibrous proteins like collagen that provides strength. Other parts are adhesive glycoproteins like fibronectin and laminin, which have a protein core with attached sugars that resist compression [20]. These proteins are assembled from recurring domains and stabilized through disulfide bonds, N‐linked glycans, and multivalent protein–protein interactions [21]. These proteins are synthesized through a secretory pathway and move through the endoplasmic reticulum and Golgi apparatus. This allows for posttranslational modifications, including formation of disulfide bonds and glycosylation, and confer the final structure and function [22, 23]. Beyond providing structural scaffolding, the ECM actively engages cell‐surface receptors, facilitating cell adhesion and bidirectional communication between cells and their environment [24]. Understanding the structure of these proteins is therefore fundamental to elucidating cell communication, immune function, and mechanotransduction, with direct implications for disease pathogenesis [25].

## 3 Biological Functions

**FIGURE 1:**
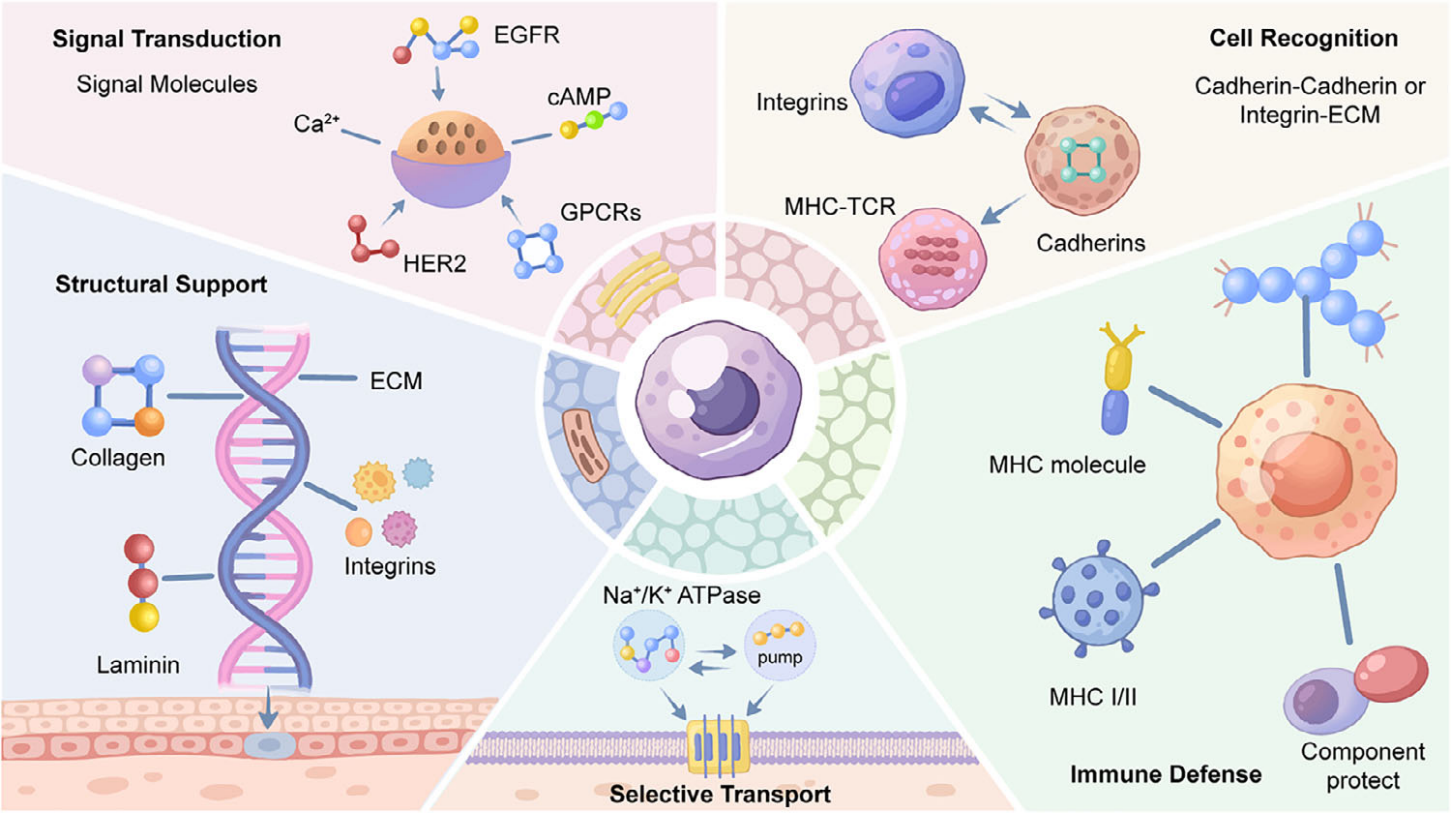
Schematic overview of the biological functions of extracellular and membrane proteins. The schematic highlights how these two proteins collectively enable critical processes: signal transduction (via GPCRs and RTKs), structural support (via collagen and laminin), selective transport (via Na^+^/K^+^ channel), cell recognition (via intergrins and cadherins), and immune defense (via MHC proteins).

Multicellular life evolved many complex systems of intercellular communication, transport systems, adhesion molecules, structure‐forming proteins, and immune defenses [26] (Figure 1). Membrane and extracellular proteins emerged as key mediators of these processes, enabling cells to sense and respond to their environment in a coordinated manner. Membrane proteins are embedded in a cell's membrane and are presently functioning as our cells’ “sensors” and “gatekeepers” [27]. Differently, extracellular proteins are those that are secreted from cells into their environment and are present as a “structural framework” and a “communication network” to organize cells [28]. Together, these proteins function to provide a “communicating system” with billions of cells functioning together to form a living organism.

### 3.1 Signal Transduction

Cells are in constant communication with their environment to survive. They could detect external signals such as hormones or growth factors and then produce the corresponding response inside [29]. This process is called signal transduction. It was enabled by both extracellular and membrane proteins. They are the cell's sensors, translators, and signal amplifiers [30]. Membrane receptors initiate signaling cascades: GPCRs undergo conformational changes to activate intracellular G proteins, receptor tyrosine kinases (RTKs) dimerize and autophosphorylate to create docking sites for downstream effectors, and ligand‐gated ion channels rapidly alter membrane potential [31]. Each type of receptor converts an external cue into a specific internal instruction, and then initiates a cascade of change in proteins that spreads and amplifies the initial stimulus. Extracellular proteins also participate in signal regulation beyond simple ligand transport. Growth factors and other signaling molecules are sequestered within the ECM and can be released upon demand, providing spatial and temporal control of signaling activity [32].

### 3.2 Selective Transport

Precise control of molecular transport across cellular membranes is essential for maintaining homeostasis [33]. This is achieved by special proteins embedded in the membrane, also known as gates, pumps, and tunnels [34, 35]. It is carried out by two types of transport proteins: channels or transporters. A channel resembles a tunnel and facilitates rapid diffusion of specific ions or molecules across membrane [36]. Transporters undergo conformational changes to translocate bound substrates across the membrane, either passively or actively (coupled to energy sources such as ATP hydrolysis) [37, 38]. Extracellular protein secretion represents a specialized transport process. Proteins destined for secretion are synthesized intracellularly, packaged into vesicles, and released into the extracellular space. This pathway mediates the delivery of structural proteins such as collagen and signaling molecules such as hormones.

### 3.3 Cell Adhesion and Recognition

Multicellular organization depends on precise control of cell–cell adhesion and recognition, enabling cells to assemble into functional tissues [39]. This process is managed through communication between membrane receptors and their partners. Adhesion molecules not only physically connect cells but also transmit signals that regulate development, migration, and differentiation of cells [40]. The immune system is a representative example of how cell–cell interaction starting. Membrane proteins, like the major histocompatibility complexes (MHC) with antigens, present this antigen to the T cell receptor. In this way, self and nonself are properly identified in the immune system [41]. This recognition system, dependent on both membrane and extracellular proteins, forms the basis of adaptive immunity.

### 3.4 Structural Support

Both membrane and extracellular proteins contribute to cellular and tissue architecture. One classic example of membrane proteins is integrins. Integrins attach a cell's intracellular skeleton with the ECM and referred to as focal adhesions. These connections transmit mechanical forces, anchor cells, and maintain cellular morphology [42]. At the tissue level, the ECM provides structural integrity. A classic example of extracellular proteins is collagen. The protein has a “helix” shape that results in strong tissue fibers and allow tissues to be strong enough to withstand stretching [43]. The dynamic interplay between membrane and extracellular proteins enables cells to survive in a demanding physical environment.

### 3.5 Immune Defense

Membrane proteins participate in every aspect of immune function, including pathogen recognition, antigen presentation, and immune cell adhesion and communication [44]. The contribution of extracellular proteins includes sending messages. Also, these proteins neutralize attackers, identify targets for destruction, and control the immune system [45]. Thus, it is obvious that these proteins play a dynamic role in protecting the body against invaders. As a representative example, MHC proteins identify peptide fragments of attackers or abnormal cells and then show these fragments to the T‐lymphocytes, initialing immune defense [46]. Membrane proteins identify attackers or threats, allowing the body to evade self‐attacks while offering short‐term or long‐term protection [47].

## 4 Dysregulation and Disease Pathogenesis

Biological systems depend on precise regulation of the synthesis, localization, and activity of membrane and extracellular proteins. These proteins operate within tightly controlled networks, with regulation occurring at genomic, transcriptional, translational, and posttranslational levels. Dysregulation significantly affects the regular functions of the protein, and this sends cells and tissues into a chain reaction, ultimately resulting in disease [48]. Therefore, it is important to understand the dysregulatory pathways, which illustrate the onset of disease and give us important insights on the development of more effective treatments.

### 4.1 Cancer

Cancer is a disease of cell's uncontrolled division and is rooted in disrupted cellular communication [49]. This pathological dialogue is driven by the dysregulation of membrane and extracellular proteins, since these problems corrupt the signaling, adhesive, and structural networks that maintain tissue homeostasis [50]. Membrane receptors and their ligands are particularly prominent in oncogenesis. For example, cancer‐causing mutations in receptors like EGFR, HER2, and c‐Met are always in an overactive state. They send signal for growth and survival constantly, without needing a trigger [51, 52]. Also, overexpression of ligands like VEGF, EGF, and TGF‐β happen during the process. This creates self‐fueling signaling loops that help tumors grow and build new blood vessels [53, 54]. Additionally, loss of adhesion molecules including E‐cadherin enables detachment from primary tumors, facilitating invasion and metastasis [55].

### 4.2 Neurological Disorders

The function of the nervous system critically depends on the precise activity of membrane and extracellular proteins [56]. These proteins maintain proper ion homeostasis, provide for neurotransmitter signaling, support synaptic connections, and maintain the integrity of the blood–brain barrier (BBB) [57, 58, 59]. When this system is disrupted, it causes serious neurological disorders. Many problems that occur within cells are because of dysfunctional membrane proteins [60]. For example, diseases called channelopathies arise from broken ion channels [61]. Mutations in the sodium or potassium channels can render neurons overly excited [62]. Extracellular protein dysfunction is equally consequential. For example, misfolded proteins, such as amyloid‐β, accumulate in Alzheimer's disease. These misfolded proteins form toxic aggregates that disrupt synapses and induce neuronal death [63].

### 4.3 Cardiovascular Diseases

Cardiovascular system depends on the regulation level of membrane proteins and extracellular proteins, which control electrical signals for heartbeat as well as vessel structures [64]. When they are not working well, it may lead to serious health problems such as arrhythmia, heart failure, atherosclerosis, and hypertension [65]. Malfunctioning membrane proteins are common as a reason for all these problems. For example, malfunctioning in β‐adrenergic receptors tends to cause problems such as heart failure. Malfunctioning in Angiotensin II receptors tends to lead to high blood pressure [66]. Both can cause harmful thickening and stiffening of heart tissue and blood vessels. Extracellular proteins are equally significant. Abnormal amounts of extracellular proteins like collagen and elastin make the heart muscle and blood vessels hard to stretch [67]. This process can be controlled by enzymes called matrix metallopeptidases (MMPs) and their inhibitors [68, 69].

## 5 TPD—An Emerging Drug Discovery Modality

Dysregulation of extracellular and membrane proteins drives numerous diseases, including cancer (overactive receptors), neurodegeneration (misfolded proteins), and cardiovascular disease (matrix remodeling). Conventional therapies often just try to block these proteins, but this approach faces significant limitations: acquired drug resistance, inability to target “undruggable” proteins, and transient effects. Moreover, intracellular degradation systems such as PROTACs cannot address extracellular or membrane proteins, creating an urgent need for alternative strategies. TPD approach lies in a paradigm shift from protein inhibition to protein elimination. There are three main strategies, including degradation of extracellular and membrane proteins by recruitment of cell‐surface LTRs, degradation of membrane proteins by recruitment of intracellular proteins, and degradation of extracellular and membrane proteins occurs independently of specific surface receptors and intracellular proteins.

### 5.1 Degradation of Extracellular and Membrane Proteins by Recruitment of Cell‐Surface LTRs

**FIGURE 2:**
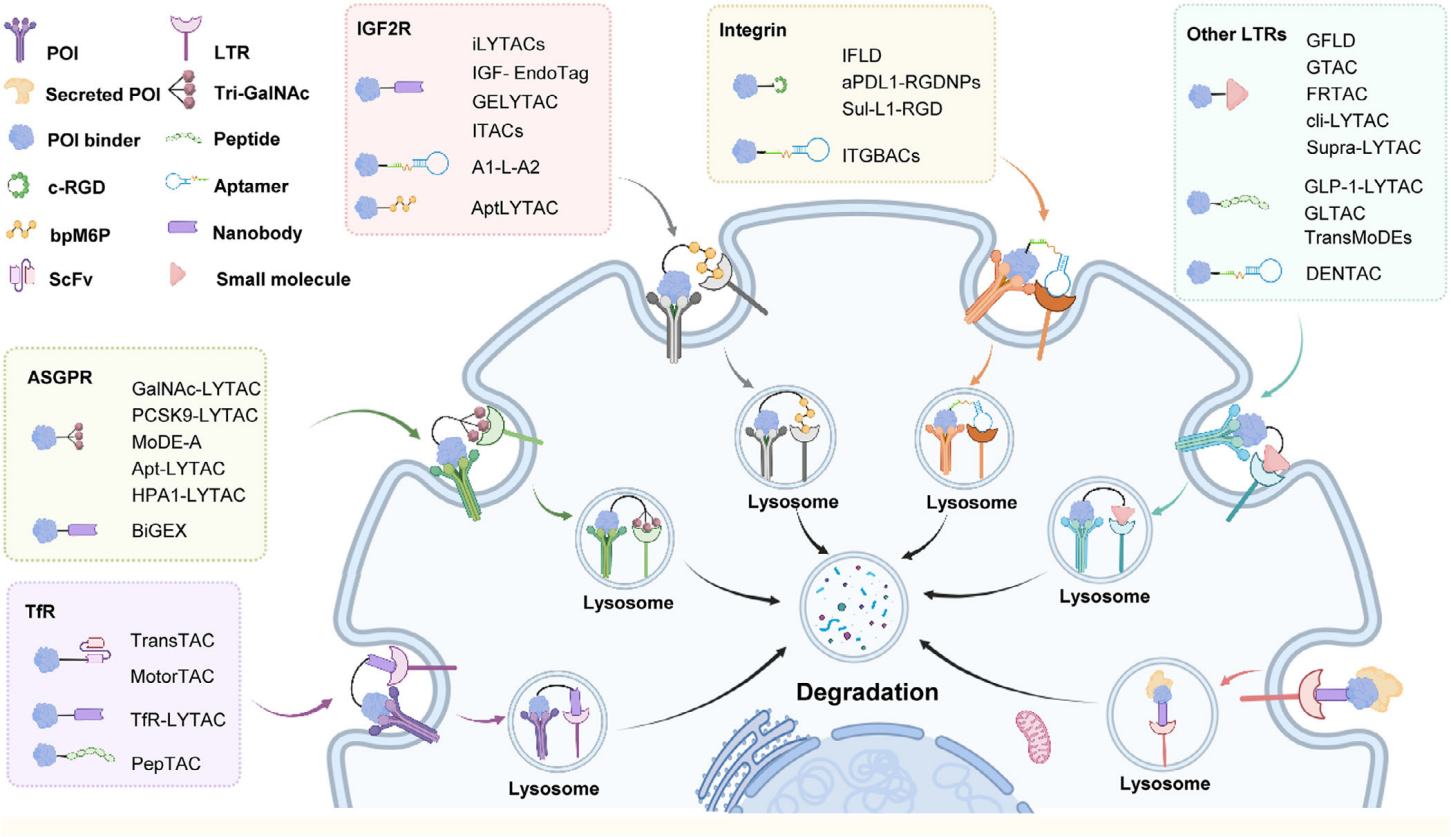
Schematic illustration of degraders that recruit various LTRs. The bifunctional degraders comprise three components: a LTR binder (such as antibody, nanobody, aptamer, and small molecule), a POI binder (such as antibody, nanobody, scFv, aptamer, and small molecule), and a linker. This complex facilitates lysosomal trafficking by physically linking the POI to the LTR, which internalizes the bound target and shuttles it to lysosomes for proteolytic clearance.

LTR‐mediated degradation relies on formation of a ternary complex comprising a bifunctional degrader, a cell‐surface LTR, and the protein of interest (POI). This complex facilitates lysosomal trafficking by physically linking the POI to LTR, which internalizes the bound target and shuttles it to lysosomes for proteolytic clearance (Figure 2). Multiple LTRs have been harnessed to enable this process, each offering unique targeting advantages. These include nutrient transporters, such as the transferrin receptor (TfR), cation‐independent mannose‐6‐phosphate receptor (CI‐M6PR), and asialoglycoprotein receptor (ASGPR); metabolic regulators, including glucose transporter (Glut1) and folate receptor (FR), which enable tissue‐specific targeting; and adhesion/signaling mediators like integrins and CD206 (macrophage mannose receptor), which broaden applications to immune cells. The diversity of LTRs provides a versatile toolkit for degrading extracellular and membrane proteins across biological contexts. For example, TfR1‐mediated degradation capitalizes on iron metabolism pathways, while CI‐M6PR leverages glycan‐based recognition. Such mechanistic variety allows researchers to tailor degraders to specific tissues, disease states, or pharmacokinetic requirements, significantly expanding the therapeutic application of extracellular and membrane protein degradation.

#### 5.1.1 TfR as the LTR

TfR plays a vital role in mediating cell iron uptake, promoting iron homeostasis under physiological conditions [70]. It is expressed at low levels in most human normal cells, but highly upregulated in cancer cells to supply sufficient iron necessary for rapid cell division [71]. The overexpression is a result of the TfR binding with transferrin, an essential protein in maintaining cancer cell growth, thus made it both a diagnostic target and a possible therapeutic target [71, 72]. Moreover, TfR undergoes continuous recycling between the cell surface and lysosomes and is widely distributed across various tissues. In recent years, TfR has emerged as a prime target for LYTACs in oncology, offering key advantages such as its frequent overexpression in tumors, which enables selective targeting of cancers while sparing healthy tissue; its efficient lysosomal trafficking, which facilitates rapid internalization and degradation of POIs; and the availability of well‐characterized, high‐affinity peptides and antibodies that have been optimized for therapeutic use [73]. These advantages make TfR a promising tool for LYTAC therapies, especially for cancers that depend on altered iron metabolism to grow. The table below summarizes the TfR‐mediated TPD strategies, detailing their molecular design, applicable target types, relevant TfR expression profiles, and reported degradation efficiencies (Table 1).

**TABLE 1:** Summary of TfR‐mediated TPD strategies.

Name	TfR binder	POI binder	Connection mode	Targets	Target types	Cell line	TfR expression (nTPM)	*D*_max_ (%)	References
TfR–LYTAC	Nanobody	ScFv	Fused expression	PD‐L1	Membrane	CT26	/	60% (100 nM)	[74]
						HCT116	184.9	50% (100 nM)	
						B16F10	/	50% (100 nM)	
TransTAC	ScFv	ScFv	Fused expression	PD‐L1	Membrane	MDA‐MB‐231	192.3	85% (10 nM)	[75]
				EGFR	Membrane	A549	138.7	90% (100 nM)	
				CD‐20	Membrane	Raji	82.4	98% (100 nM)	
				CAR	Membrane	Jurkat	63.7	80% (100 nM)	
MotorTAC	ScFv	Aptamer	Connected by NPs	PDGF	Extracellular	SiHa	173.6	91% (10 nM)	[76]
Pep‐TAC	Peptide	Peptide	Solid phase synthesis	PD‐L1	Membrane	B16	/	78% (25 µM)	[77]
						MC38	/	70% (25 µM)	
						DC2.4	/	54% (25 µM)	
						RAW264.7	/	69% (25 µM)	

In 2024, Nie et al. developed a modular, genetically encoded lysosome‐targeting chimera (TfR–LYTAC) by exploiting the rapid endocytic cycle of the TfR [74]. This platform integrates two key domains within a single gene construct: a TfR‐binding peptide and the single‐chain variable fragment (scFv) from avelumab, an United States Food and Drug Administration‐approved anti‐PD‐L1 antibody [78]. Microscale thermophoresis assays confirmed the construct's high binding affinity for both PD‐L1 (*K*_d_ = 52 nM) and TfR (*K*_d_ = 21 nM). In cells, the TfR–LYTAC successfully directed PD‐L1 to the lysosome for degradation [79]. This was confirmed in multiple cancer cell lines. But in a mouse tumor model, the treatment did not suppress tumor growth obviously, likely due to poor plasma stability and tissue retention of the peptide‐based construct. To address this, the researchers developed outer membrane vesicle (OMV)‐based delivery by fusing TfR–LYTAC to the ClyA protein embedded in OMVs with an MMP‐cleavable linker [74]. This new system, named OMV–LYTAC, aims to combine targeted degradation with immune stimulation. However, the efficiency of PD‐L1 degradation in vivo is still low. Future work needs to improve the stability of the system, pharmacokinetic, and its activation at the tumor site to make it more effective.

TransTACs represent a novel class of bispecific antibodies that exploit the TfR pathway for TPD [75]. Their design includes a synthetic antibody part and a cleavable linker, directing membrane proteins to lysosome for degradation. In vitro, it was found that TransTACs had shown incredible potential and effectiveness by degrading over 80% of POIs, including immune checkpoint (PD‐L1), oncogenic driver (EGFR), and therapeutic target (CD20). In animal models, TransTACs significantly suppressed tumor growth in EGFR‐mutant lung cancer models. TransTACs combine advantages of multiple degradation platforms: broader target scope than PROTACs, enhanced tumor selectivity due to TfR overexpression in cancers, and modular design facilitating adaptation to new targets.

Conventional targeted therapies often depend on passive diffusion, resulting in inefficiency in biological environments [80]. To increase the potency of targeted therapies, MotorTACs are developed as a different platform. They use self‐propelled nanomotors to actively seek for targets. They were designed to degrade extracellular proteins like PDGF [76]. A MotorTAC contains four parts: an aptamer to recognize the POI, a nanomotor for movement, transferrin to hijack the TfR pathway, and a E3 ligand to recruit the E3 ligase and UPS system [81]. This platform enables dual degradation: the POI is internalized via TfR and simultaneously tagged by E3 ligase for proteasomal destruction. Movement of the nanomotor accelerates its path toward the targets, achieving internalization within 3 h and complete degradation within 4 h. This platform unlocks a new direction for the degradation of extracellular proteins by combining multiple technologies.

Another strategy seeks to expand upon a basic TfR‐based LYTAC. The initial construct consists of a peptide conjugated to an PD‐L1 antibody. However, this system has important limitations. The peptide degrades rapidly, and PD‐L1 degradation was incomplete and transient, likely due to a noncovalent interaction with TfR. Efforts addressing these deficiencies led to the development of covalent tagging methods. One such technology involves sulfur (VI) fluoride exchange reactions [82]. Aryl sulfonyl fluorides (ASFs) exhibit a broad reactivity and biocompatibility to target a wide range of nucleophilic residues, including Lys, Tyr, and His [83, 84, 85]. Building on this, Gao et al. developed Pep‐TACs, which uses a covalent chemistry named SuFEx [77]. It forms a stable, irreversible bond with the target protein PD‐L1 enhancing the binding affinity. It also has a TfR‐targeting peptide. This means the target is tightly captured and efficiently taken to the lysosome. Pep‐TACs caused strong and lasting PD‐L1 degradation in cells. Notably, Pep‐TACs significantly inhibited tumor growth and extended the survival of mice in orthotopic brain tumor models, achieving a 50% tumor regression rate. Moreover, they were able to cross the BBB, and their modular design could be adapted for other targets.

#### 5.1.2 ASGPR as the LTR

Many LYTAC platforms are limited by broad LTR expression across human tissues, resulting in off‐target effects in healthy tissues. ASGPR is expressed exclusively on hepatocytes, making it ideal for liver‐specific degradation [86]. Its physiological role involves clathrin‐dependent uptake of glycoproteins that have GalNAc or galactose at their terminals [87]. Following internalization, acidic endosomal pH promotes ligand release, enabling ASGPR recycling to the cell surface. This recycling pathway renders ASGPR particularly suitable for liver‐specific TPD. It has clear benefits: it targets only liver cells, so degradation stays in the liver; it clears proteins well in terms of the liver's natural breakdown system for efficient protein removal; and it is flexible because scientists can attach synthetic glycoside ligands (like GalNAc) to different degraders [88, 89, 90]. The table below shows ASGPR‐mediated TPD strategies, their design, target types, ASGPR expression, and degradation efficiencies (Table 2).

**TABLE 2:** Summary of ASGPR‐mediated TPD strategies.

Name	ASGPR binder	POI binder	Connection mode	Targets	Target types	Cell line	ASGPR expression (nTPM)	*D*_max_ (%)	References
GalNAc–LYTAC	Small molecule	Antibody	Chemical conjugation	EGFR	Membrane	HEP3B	340.9	70% (10 nM)	[91]
						HepG2	566.4	61% (10 nM)	
						HUH7	150.5	52% (10 nM)	
				HER2	Membrane	HepG2	566.4	75% (100 nM)	
PCSK9–LYTAC	Small molecule, antibody	Small molecule, antibody	Chemical conjugation	PCSK9	Extracellular	HEK293	Over expressed	/	[92]
MoDE‐A	Small molecule	Small molecule	Chemical synthesis	α‐DNP	Extracellular	HepG2	566.4	/	[93]
				MIF	Extracellular			/	
BiGEX	Nanobody	Nanobody	Coexpressed on the exosome surface	PD‐L1	Membrane	HepG2	566.4	50% (0.2 g/L)	[94]
				HER2	Membrane			/	
Apt–LYTAC	Small molecule	Aptamer	Solid phase synthesis	PDGF	Extracellular	HepG2	566.4	59% (50 nM)	[95]
				PTK7	Membrane			31% (500 nM)	
HPA1–LYTAC	Small molecule	Small molecule	Chemical synthesis	HPA1	Extracellular	HepG2	566.4	70% (2 µM)	[96]

The high‐affinity interaction between the ASGPR and triantennary N‐acetylgalactosamine (tri‐GalNAc) has been successfully exploited in drug delivery systems, with several candidates currently in clinical development [97]. Building on this foundation, researchers have adapted the ASGPR/tri‐GalNAc system for TPD. In 2021, Bertozzi et al. created GalNAc‐conjugated LYTACs (GalNAc–LYTACs) by conjugating antibodies to a tri‐GalNAc ligand that engages ASGPR to direct bound proteins to lysosomes [91]. The platform was validated using cetuximab (CTX) (anti‐EGFR) and pertuzumab (anti‐HER2) conjugated to tri‐GalNAc. These constructs achieved robust, ASGPR‐dependent degradation (70–75%) of their respective membrane protein targets in hepatoma cells, significantly outperforming the inhibitory effects of antibodies alone and leading to sustained pathway attenuation. GalNAc–LYTACs thereby underscored the modularity of the platform for more POIs and brought several advantages: hepatocyte‐specific targeting via the liver‐restricted expression of ASGPR, improved pharmacokinetics wherein site‐specific tri‐GalNAc conjugation enhances its stability and plasma half‐life, and superior efficacy due to the fact that degradation outperforms traditional antibody inhibition. In parallel, Tang et al. expanded the platform's versatility by creating tri‐GalNAc conjugates with biotin, antibodies, or antibody fragments to generate a novel class of degraders [98]. They demonstrated that extracellular protein (like antibiotin IgG‐647) could be successfully internalized into cells and sent to lysosomes for degradation in liver cells. This work shows GalNAc–LYTACs are a good tool for addressing TPD to specific cell types.

Proprotein convertase subtilisin/kexin Type 9 (PCSK9) is a key regulator of cholesterol metabolism, and it causes degradation of the hepatic low‐density lipoprotein (LDL) receptor (LDLR). Current treatments include antibody (like alirocumab and evolocumab) that stop PCSK9 from binding to LDLR, and siRNA that reduce PCSK9 production [99]. However, these treatments have limitations in oral bioavailability and large‐scale production. To address this, the Clairmont et al. made a PCSK9–LYTAC by attaching a tri‐GalNAc molecule to a PCSK9 ligand and used the liver cell‐specific ASGPR pathway [92]. This approach was tested with different antibody and small‐molecule formats, and it showed accelerated PCSK9 clearance in vivo. Interestingly, unlike small molecules, a bifunctional degrader of PCSK9 has its advantages, such as enhanced clearance by catalytic degradation and the potential for oral delivery and scaled‐up synthesis. Though this holds promise, its mechanism must be further characterized, including using non‐ASGPR models and finding out whether this pathway can act independently.

The ASGPR has parts with C‐type lectin domains that bind galactose derivatives like GalNAc [100]. After the ligand binds and enters the cell, the acidic area inside the endolysosome causes the ligand to release, so ASGPR can recycle and shuttle the POIs goes to lysosomes for degradation [101]. Spiegel et al. exploited this pathway to develop molecular degraders of extracellular proteins via ASGPR (MoDE‐As) [93]. These molecules comprise an ASGPR‐targeting motif, a polyethylene glycol (PEG) spacer, and a target protein binder. MoDE‐As facilitate ternary complex formation between ASGPR and the POI on the liver cell surface, driving internalization and lysosomal degradation of diverse extracellular targets, like antibodies and cytokines, both in vitro and in vivo. MoDE‐As offer clear benefits over other TPD technologies, such as liver‐specific clearance using liver's high breakdown capacity; immune tolerance, helped by ASGPR's role in keeping immune balance; and a wider target range, tested on proteins from circulating antibodies to pathogenic aggregates. This platform shows a successful utilization of receptor biology to achieve tissue‐limited protein degradation.

Current TPD strategies face problems from chemical synthesis of chimeric molecules, poor in vivo delivery, body‐wide spread barriers, and dose‐limiting side effects. To address these limitations, advanced delivery systems have been tried, such as nanoparticles (NPs), polymers, and viral vectors. Among these, exosomes have appeared as a very promising platform because they are naturally biocompatible, have low immune reactions, and can be precisely engineered [102]. Early work by Zhang et al. showed the potential of engineered exosomes in immunotherapy, and they made bispecific exosomes (like aEGFR/aCD3) that link cancer cells and T cells to boost antitumor immunity [103]. Building on this delivery concept, He et al. made a TPD‐specific platform: bispecific guided exosomes (biGEX) [94]. These exosomes are made to show scFvs against an LTR (like ASGPR) and a POI (like PD‐L1 or HER2) on their surface. The biGEX degraded PD‐L1 and HER2 in HepG2 cells, proving ASGPR‐mediated lysosomal sorting. Exosome‐based TPD platforms like biGEX show a novel and strategic approach, and they address key limitations in conventional degrader delivery. But moving these findings to clinical applications requires overcoming challenges in scalable production of genetically engineered exosomes and doing systematic in vivo efficacy and safety studies.

Aptamers are single‐stranded DNA/RNA that form specific shapes, so they can bind strongly and specifically to different targets, including proteins, small molecules, and cells. These kinds of DNA/RNA, often called “chemical antibodies,” have clear benefits over conditional drugs, such as cheap production through in vitro SELEX; low immune reactions, so they are ideal for treatment; and flexible structure, because they are easy to change at specific sites for conjugation or labeling [104, 105, 106]. But aptamers have limits in modulating protein activity: targeting active sites and partial blocking. TPD technologies address these problems by removing the target protein completely, so they allow functional changes even with weak binders that do not target active sites [107]. In 2023, Zhu et al. developed aptamer‐based LYTACs (Apt–LYTACs) by attaching protein‐specific aptamers to tri‐GalNAc, and they used the liver cell‐specific ASGPR to degrade both extracellular and membrane proteins through lysosomal transport [95]. These Apt–LYTACs degrade extracellular proteins like PDGF and membrane proteins like PTK7 quickly in HepG2 cells through a lysosomal degradation pathway. Above all, this approach has several key features, including flexibility from switching aptamer modules to allow targeting of different proteins; efficiency, due to the smaller size improving cell entry and lysosomal movement; and scalability, because of a cheap and repeatable production process suitable for high‐throughput screening.

NK cells are critical for immune defense against liver cancer (HCC), functioning through interactions between natural cytotoxicity receptors (NCRs) and their heparan sulfate (HS)‐containing ligands [108, 109]. Heparanase (HPA1), an enzyme overexpressed in HCC, cleaves HS chains on NCR ligands, enabling tumor immune evasion [110]. High HPA1 levels correlate with aggressive HCC features, like microvascular invasion and multifocal tumor growth [111]. LYTACs targeting proteins involved in atherosclerosis (like PCSK9) or immune checkpoints (like PD‐L1) have shown a TPD success, but their use in immunology research is still limited [92]. To stop HPA1‐driven immune escape, Wang et al. made JW‐9, which is a small‐molecule LYTAC made of a tri‐GalNAc and an HPA1 inhibitor [96]. JW‐9 forms a ternary complex with HPA1 and ASGPR on hepatocytes. Then, the complex undergoes clathrin‐mediated endocytosis, followed by lysosomal degradation of HPA1. Due to its small size and lack of antigen‐binding site variability, JW‐9 effectively degrades HPA1 and then restores HS‐modified NCR ligands on HCC cells, enhancing the recognition and cytotoxicity of NK cells both in vitro and in vivo. HPA1–LYTAC represents a significant innovation with three key implications: it constitutes the first small‐molecule LYTAC for extracellular proteins, overcoming the size and immunogenicity limitations of antibody‐based platforms; it marks the first application of LYTAC technology to enhance innate immune responses, thereby bridging the fields of TPD and cancer immunotherapy; and it validates HPA1 inhibition as a viable strategy to boost NK cell immunosurveillance, offering a novel therapeutic approach for HCC treatment. Overall, JW‐9 exhibits the transformative potential of small‐molecule LYTACs. By restoring NK cell function through HPA1 elimination, this strategy addresses a critical unmet need in HCC treatment and establishes a blueprint for extracellular protein‐targeted immune modulation.

#### 5.1.3 Integrin as the LTR

Due to the liver‐specific expression of ASGPR, the application of ASGPR‐mediated LYTACs is inherently restricted to live cancers. To broaden the therapeutic scope of TPD to other cancer types, there is a compelling need to exploit alternative receptors that are ubiquitously overexpressed in many cancers. Integrins are cell adhesion receptors that are highly expressed in many cancers and are linked to tumor growth, spread, and blood vessel formation [112, 113]. Their overexpression in malignancies positions them as ideal targets for selective degradation of POIs. The arginine–glycine–aspartic acid (RGD) motif is a canonical integrin‐binding sequence, and cyclization of RGD (cRGD) enhances stability, binding affinity, and specificity, making it a robust tool for cancer targeting [114]. cRGD‐functionalized NPs have demonstrated efficient tumor accumulation via integrin‐mediated endocytosis, suggesting its utility in LYTAC design [115]. A proposed integrin‐based LYTAC platform consists of a cRGD‐based targeting moiety linked to a POI binder. Upon binding, the integrin–LYTAC complex undergoes receptor‐mediated endocytosis, shuttling the POI to lysosomes for degradation. This approach exploits integrins’ natural endocytic recycling, bypassing ASGPR's hepatic restriction. The table below summarizes key integrin‐mediated TPD strategies, including the design details of key components, applicable target types, the integrin expression profiles, and maximum degradation efficiencies in each technology (Table 3).

**TABLE 3:** Summary of integrin‐mediated TPD strategies.

Name	Integrin binder	POI binder	Connection mode	Targets	Target types	Cell line	Integrin expression (nTPM)	*D*_max_ (%)	Referennces
IFLD	Peptide	Small molecule	Chemical synthesis	PD‐L1	Membrane	MDA‐MB‐231	41.9	75% (25 nM)	[116]
aPDL1–RGD NPs	Peptide	Antibody	Chemical conjugation	PD‐L1	Membrane	B16F10	/	85% (50 nM)	[117]
Sul‐L1–RGD	Peptide	Small molecule	Chemical synthesis	CAIX	Membrane	MDA‐MB‐231	41.9	50% (5 nM)	[118]
ITGBACs	Aptamer	Aptamer	Enzymatic synthesis	CD71	Membrane	DU‐145	24.6	87% (500 nM)	[119]
				PTK7	Membrane	HCT116	21.2	89% (800 nM)	

In 2022, Fang et al. developed integrin‐facilitated lysosomal degradation (IFLD), employing bifunctional molecules comprising target‐binding ligands connected to cRGD motifs [120]. IFLD uses integrin‐mediated uptake to move POIs into the endosomal–lysosomal path, bypass using liver receptors like ASGPR. As a proof‐of‐concept, they demonstrated that biotin–RGD enhanced the cellular uptake and lysosomal degradation of NeutrAvidin and other POIs, indicating the platform's broad applicability [121]. Then they expanded IFLD on the important immunotherapy target PD‐L1. Considering BMS‐8 is a small‐molecule PD‐L1 inhibitor, they prepared a degrader (BMS‐L1–RGD) with a variable‐length PEG spacer to improve the ternary complex formation between the degrader, PD‐L1, and αvβ3 integrin [116]. Furthermore, they found that the degradation depends on lysosomes, because bafilomycin A1 blocked it but the proteasome inhibitor MG132 did not. In a xenograft model, BMS‐L1–RGD reduced tumor size by 60% compared with controls and exhibited minimal off‐target toxicity. This demonstrates the therapeutic benefit of integrin's cancer‐selective overexpression. BMS‐L1–RGD offers several advantages as a protein degrader, including precision, achieved by leveraging integrin overexpression in tumors for selective degradation; scalability, due to a design adaptable to diverse POIs via changing ligands for recognizing targets; and favorable pharmacokinetics, with enhanced tissue penetration and potential for oral bioavailability.

LYTACs are a new way to address the limitations of conventional inhibitors, especially for immune checkpoint proteins like PD‐L1. While conjugated or engineered antibodies have shown clinical promise, their effectiveness is often limited by short‐term blocking and incomplete elimination of POIs. To address this, recent efforts have harnessed LTRs, which are highly expressed in tumors, to enhance TPD. In 2025, Tang et al. developed a TPD platform by attaching antibodies to cRGD peptides [117]. Multivalent RGD peptides improve targeting efficacy through receptor clustering, enhancing both binding avidity and uptake efficiency compared with monomeric RGD [122]. This principle was applied to make aPD‐L1–RGD NPs, made of poly(glutamic acid) backbones with multivalent RGD peptides and PD‐L1 antibodies [123]. Multivalent RGD binds αvβ3 integrins, causing receptor‐mediated uptake and then lysosomal degradation of PD‐L1. This process removes PD‐L1 and stops its recycling to the cell surface, keeping T cell activation by preventing immune checkpoint rescue. In preclinical models, aPD‐L1–RGD NPs worked better than standard PD‐L1 antibodies, causing a big increase in intratumoral pro‐inflammatory cytokines (IFN‐γ, TNF‐α, granzyme B). This strong immune activation was achieved to enhance cytotoxic T cell responses and tumor suppression.

Solid tumors develop hypoxic and acidic microenvironments caused by abnormal metabolism. A key factor in this adaptation is carbonic anhydrase IX (CAIX). It is an enzyme induced by low oxygen and it controls tumor pH to facilitate survival, invasion, and metastasis [124]. While CAIX inhibitors like SLC‐0111 (currently in clinical trials) transiently block enzymatic activity, targeted degradation of CAIX offers a more durable strategy to disrupt tumor adaptation. To achieve this, Chen et al. made a new type of bifunctional degraders by conjugating CAIX‐specific ligands to cRGD peptides with linkers of different lengths [118]. These compounds target both αvβ3/αvβ5 integrins overexpressed on tumor cells and CAIX on tumor cell membranes. The double binding facilitates integrin‐mediated uptake and lysosomal degradation of CAIX, and it disrupts its pH‐regulatory function. Among synthesized compounds, Sul‐L1–RGD was the lead one, and it achieved ∼50% CAIX degradation at the cellular level. Incomplete degradation efficiency may come from CAIX's two locations (membrane and nucleus), which limits lysosomal access for nuclear areas. This drug demonstrated potent antiproliferative effects in hypoxic conditions: 60% reduction in HT‐29 cell survival and 55% survival inhibition in MDA‐MB‐231. By removing CAIX rather than temporarily blocking it, this approach disrupts the tumor's adaptive machinery and reduces the tumor's ability to survive in harsh microenvironments.

Aptamers are seen as flexible alternatives to antibodies in diagnostics and treatments [125]. Their programmability, low immunogenicity, and ease of preparation make them ideal for innovative drug delivery systems. Tan et al. exploited this potential when they found DML‐7. It is a DNA aptamer that binds strongly to DU145 prostate cancer cells, and it was later found to bind integrin α3β1 (ITGA3β1). They found that DML‐7 entered cells through receptor‐mediated uptake, and they thought ITGA3β1 might be used for TPD. So they developed integrin‐binding aptamer chimeras (ITGBACs) [119]. The ITGBAC platform uses two aptamers. One targets Integrin and the other one targets a membrane‐bound protein, such as CD71 or PTK7. When both receptors were linked at the same time, this triggers cell uptake and lysosomal transport, and then results in degradation of the target proteins. This approach leads to cause lysosomal degradation of CD71 in prostate cancer cells and PTK7 in colorectal cancer cells. Also, in DU145 xenograft models, ITGBACs suppressed tumor growth without inducing systemic toxicity. Compared with conventional TPD strategies, ITGBACs have several key advantages. They are modular because new aptamers targeting different proteins can be integrated via Cell‐SELEX. This avoids traditional antibody engineering problems. They have tumor selectivity because they use ITGA3β1 overexpression in tumors to reduce off‐tissue effects. And they can be scalable because their DNA‐based synthesis allows cost effective and rapid production compared with protein‐based systems.

#### 5.1.4 IGF2R/CI‐M6PR as the LTR

CI‐M6PR (also known as IGF2R) is a protein naturally found in mammalian cell membranes [126, 127]. It has two roles: it moves N‐glycosylated proteins marked with mannose‐6‐phosphate (M6P) to lysosomes, which is important for maintaining cellular homeostasis; also, it modulates IGF2 levels through high‐affinity binding (*K*_d_ ≈ 40 nM), so it affects cell growth and metabolism signals [128, 129]. These properties have been exploited in cancer therapy, and M6P‐attached drugs (like enzymes and peptides) are delivered through CI‐M6PR‐mediated endocytosis. The advent of LYTACs further expanded its utility, enabling targeted degradation of extracellular and membrane proteins by forming ternary complexes (CI‐M6PR–LYTAC–POI) that route POIs to lysosomes [130]. The table below summarizes IGF2R/CI‐M6PR‐mediated TPD strategies, including the design details of key components, applicable target types, the IGF2R/CI‐M6PR expression profiles, and maximum degradation efficiencies in each technology (Table 4). Despite progress, CI‐M6PR‐focused approaches face critical barriers, including complicated glycan synthesis, where generating M6P glycans involves multistep chemical or enzymatic processes, suffer from low yields and poor scalability; product heterogeneity, as conjugating glycans to antibodies or ligands via chemical linkers complicate reproducibility and regulatory approval; and antibody engineering bottlenecks, which require laborious optimizations. These limitations hinder the clinical translation of CI‐M6PR‐dependent degraders, underscoring the need for innovative TPD tools.

**TABLE 4:** Summary of IGF2R/CI‐M6PR‐mediated TPD strategies.

Name	IGF2R binder	POI binder	Connection mode	Targets	Target types	Cell line	IGF2R expression (nTPM)	*D*_max_ (%)	References
iLYTACs	Nanobody	Nanobody/antibody	Fused expression	EGFR	Membrane	HeLa	30.1	71% (200 nM)	[131]
				PD‐L1	Membrane	MDA‐MB‐231	21.4	55% (200 nM)	
			Z domain/Fc	CD20	Membrane	Raji	9.0	70% (100 nM)	
				α‐Syn	Extracellular	K562	29.4	20% (200 nM)	
IGF‐EndoTag	Nanobody	Nanobody	Fused expression	EGFR	Membrane	H1975	30.4	64% (200 nM)	[132]
						HeLa	30.1	82% (200 nM)	
				PD‐L1	Membrane	MDA‐MB‐231	21.4	77% (200 nM)	
GELYTAC	Nanobody	Nanobody	Fused expression	IL6‐R	Extracellular	K562	29.4	/	[133]
						Primary T	/	/	
		ScFv		TGF‐β	Extracellular	K562	29.4	/	
A1‐L‐A2	Aptamer	Aptamer	Enzymatic synthesis	Met	Membrane	HeLa	30.1	88% (300 nM)	[134]
				PTK‐9		CEM	/	43% (500 nM)	
AptLYTAC	Small molecule	Aptamer	Biotin/streptavidin	PTK7	Membrane	Jurkat	27.1	94% (500 nM)	[135]
						SUM159	23.9	90% (500 nM)	
				Met	Membrane	MDA‐MB‐231	21.4	90% (500 nM)	
						SUM159	23.9	76% (500 nM)	
ITACs	Nanobody	Aptamer	Base pair	Met	Membrane	HeLa	30.1	70% (600 nM)	[136]
						DU145	18.0	70% (600 nM)	
						A431	31.0	70% (600 nM)	
				PTK7	Membrane	A431	31.0	/	
						HeLa	30.1	62% (600 nM)	
				EpCAM	Membrane	A431	31.0	54% (600 nM)	
				FGFR2	Membrane	A431	31.0	38% (600 nM)	

In 2023, Ge et al. pioneered insulin‐like growth factor 2 LYTACs (iLYTACs), a novel class of genetically encoded degraders [131]. These chimeras fuse IGF2, a natural 6.5‐kDa ligand of the insulin‐like growth factor 2 receptor (IGF2R/CI‐M6PR), to recombinant proteins targeting extracellular or membrane‐bound POIs. The platform's efficacy was rigorously validated. Binding tests confirmed that iLYTACs keep high affinity for IGF2R (*K*_d_ ≈ 3.4–65.03 nM). A key design involved fusing IGF2 to an EGFR‐targeting affibody (Af_EGFR_). This caused significant EGFR degradation. However, pretreatment with either free IGF2 or the affibody alone could rescue this degradation, confirming that a ternary complex is required in the performance of iLYTAC. Further, Type‐I iLYTACs demonstrated broad use because they degraded different targets: EGFR, PD‐L1, fibrillar α‐synuclein and CD20. For EGFR, they saw significant reduction of downstream kinase signaling after iLYTAC‐assisted EGFR elimination. They checked the antitumor properties of iLYTACs in a mouse model, where in vivo degradation of EGFR was observed. Also, they developed Type‐II iLYTACs (IGF2‐Z). This is a modular nanobody that works with off‐the‐shelf monoclonal antibodies (mAbs). Just mixing IGF2‐Z with mAbs results in TPD. So iLYTACs work as a plug‐and‐play toolbox for effective elimination of extracellular and membrane proteins. in vivo, IGF2‐Af_EGFR_ (Type‐I) and IGF2‐Z + CTX (Type‐II) worked better than CTX alone, demonstrating enhanced tumor killing for degraders. Overall, iLYTACs have several distinct advantages, which include plug‐and‐play modularity for quick adaptation to new targets; genetic engineered, avoiding the problems of bioconjugation such as glycan‐antibody linkages; and cost efficiency.

Traditional approaches to induce endocytosis and lysosomal trafficking of cell surface receptors often rely on endogenous ligands. But these methods have limitations, such as competition with native ligands and reliance on chemical modifications that hinder genetic encodability and scalable production. Furthermore, many receptors lack natural ligands capable of triggering endocytosis. To solve this, the Baker et al. created EndoTags. These are designed proteins and selectively trigger receptor endocytosis without activating downstream signaling pathways [132]. Leveraging structural insights, they hypothesized that inducing conformational changes in the IGF2R could mimic ligand‐driven endocytosis. Native ligand IGF2 binding induces IGF2R dimerization by bridging domains 6 (D6) and 11 (D11). Using the Rosetta RIFdock computational tool, the team designed compact protein binders (“minibinders”) that simultaneously engage D6 and D11, utilizing IGF2's dimerization without initiating IGF2‐related signaling [137]. They measured the binding affinities of binders by BLI and found that the tightest binder to D6 had an affinity of 41 nM and to D11 had an affinity of 6.5 nM. These engineered proteins, termed EndoTags, enable controlled lysosomal trafficking of target receptors. After optimizing these minibinders, they made genetic fusions of EndoTag1 with CTX, which is an anti‐EGFR antibody. This is named CTX–IGF_EndoTag1 [138]. In H1975 cells, CTX–IGF_EndoTag1 reached 85% EGFR degradation at 10 nM, which worked better than M6P‐based LYTACs [130]. Protein analysis confirmed that it was specific, and it had minimal effect on IGF2R levels. In the same way, when atezolizumab (ATZ) fused to EndoTag1 (ATZ–EndoTag1), it reduced PD‐L1 levels by 77% in MDA‐MB‐231 cells. Also, a CTLA4‐specific minibinder fused to EndoTag1 reduced CTLA4 levels by 45% in Jurkat cells. Importantly, in an A20 lymphoma mouse model, PD‐L1‐EndoTag1 fusions showed better tumor suppression than antibody treatment alone, indicating its therapeutic potential. This IGF‐EndoTag platform has several key advantages. It is genetically encodable, so it does not need chemical attachment and allows simple recombinant production. It has better specificity because it avoids off‐target signaling. And it is versatile, shown by its ability to adapt to different targets such as EGFR and PD‐L1 through fusion with various antibody or minibinder scaffolds.

LYTACs enable degradation of extracellular and membrane proteins by hijacking LTRs. First‐generation LYTACs used synthetic glycopeptides, but these cannot be genetically engineered or made easily in large amounts. To address this, Ting et al. made genetically encoded LYTACs (GELYTACs). This is a modular platform that combines protein engineering and directed evolution for precise, scalable extracellular protein removal [133]. GELYTACs have two modular parts: a small protein binder (like a nanobody or scFv) specific to the target protein, and a directed‐evolution‐optimized variant of IGF2. They optimized the IGF2 scaffold through directed evolution to improve its binding properties. GELYTACs connect target proteins (TGF‐β, shed IL6R ectodomain) to IGF2R, and this triggers receptor‐mediated uptake and lysosomal degradation. The Ting group validated this platform on multiple targets: a model protein (mCherry) and therapeutic targets (TGF‐β and IL6R). They found that engineered primary human T cells secreting GELYTACs, which induced tumor cell uptake of target proteins and allowed in situ degradation within the tumor environment. This approach involves the combination of cellular therapy and TPD for targeted therapy. The advantages of the GELYTAC platform lie in the fact that it can be made on a large scale by a recombinant approach, which does not require glycan synthesis; it can be flexibly modified toward POIs because it is composed of modules; and last, the platform can be delivered via mRNA, virus, or cells for in vivo delivery.

Aptamers have appeared as promising tools for degrading membrane proteins through LYTACs [135]. They are attractive because they are modular, easy to make, and work with existing aptamer libraries targeting clinically relevant proteins [139]. However, the typical Apt–LYTAC utilizes challenging chemical ligation strategies that hamper scalability and reproducibility. To address this, Han et al. prepared bispecific aptamer chimeras designated as A1‐L‐A2. This represents a fully nucleic acid‐based strategy that bypasses needing for chemical ligation while enabling accurate lysosomal degradation of POIs [134]. The A1‐L‐A2 platform has three main parts: a target‐binding aptamer (A1); an IGF2R‐targeting aptamer (A2); and a double‐stranded DNA linker (L). Linker is engineered to give optimal spatial flexibility and improve overall binding efficiency. In this approach, A1‐L‐A2 connects the target protein and IGF2R, and this triggers clathrin‐mediated uptake. Then the complex moves to lysosomes, where the target is permanently degradation. Han et al. used a bispecific aptamer named D3. It had a 23 bp ds‐DNA linker, and they used it to test Met elimination in HeLa cells. The results were promising: D3 reduced Met levels to 34% of the control. Also, they made another bispecific aptamer, D4, with a 22 bp ds‐DNA linker, to target PTK‐7 in CEM cells. D4 achieved a 57% reduction in PTK‐7 levels compared with the control. This approach offers key advantages. It has simplified synthesis because it is fully nucleic acid‐based, avoiding complicated chemical attachments; it allows rapid development because it uses prevalidated aptamer libraries for faster targeting of new POIs; and it has tumor selectivity in terms of the common overexpression of IGF2R in tumors.

Existing LYTACs degrade single proteins, but clearing multiple POIs at the same time remains a critical unmet need. To address this, Han et al. made IGF2‐tagged aptamer chimeras (ITACs), which allow codegradation of two proteins through a single LTR [136]. ITACs are antibody‐oligonucleotide conjugates (AOCs) with two main parts. First, an antibody or nanobody (like IGF2) directs lysosomal trafficking by binding the widely expressed IGF2R. Second, a combined aptamer complex is made of two different protein‐targeting aptamers connected through complementary DNA sequences. This triggers clathrin‐mediated uptake and lysosomal degradation of both targets at the same time. ITACs were shown to effectively degrade several membrane proteins, including c‐Met, PTK7, EpCAM, and FGFR2 in HeLa cells. Also, at low concentrations, bispecific ITACs worked better than the combined effect of two single‐target ITACs. They achieved over 60% codegradation of dual target proteins. They believed that the cooperative degradation effect came from two key points. First, mITACs–MET–PTK7 had higher binding affinity to HeLa cells (the *K*_d_ was 3.8 nM for mITACs–MET–PTK7, 9.9 nM for ITACs–MET, and 7.3 nM for ITACs–PTK7). Second, there was cooperativity between MET and PTK7 aptamers in mITACs–MET–PTK7 to form a more stable ternary structure. Additionally, the researchers made a universal DNA linker. This allowed precise control of aptamer amounts, and it ensured optimal spatial alignment for simultaneous target protein engagement. The ITACs platform has several distinct advantages. It can remove two targets at once to clear compensatory cancer pathways (like c‐Met and FGFR2) at the same time, reducing the risks of acquired resistance; it is modular, so it can be adapted to different POIs through simple aptamer exchange without needing to redesign the core scaffold; moreover, it has enhanced efficiency, because cooperative binding minimizes dose requirements and consequently lowers off‐target risks.

#### 5.1.5 Other LTRs

LTR‐dependent TPD strategies have expanded beyond classical receptors (like TfR, ASGPR, IGF2R) to include new effectors. Each offers unique advantages for cancer therapy. The table below summarizes other LTR‐mediated TPD strategies. It includes the design details of key components, target types, LTR expression profiles, and maximum degradation efficiencies in each technology (Table 5). These include GLUT1, FR, CD206, glucagon‐like peptide‐1 receptor (GLP‐1R), Glypican‐3 (GPC3), scavenger receptors (SRs), clathrin, and CAIX. The variety of LTRs in TPD strategies underscores the field's innovation. By using receptor biology unique to cancer cells, these approaches reduce off‐target effects and address unmet clinical needs.

**TABLE 5:** Summary of other LTRs‐mediated TPD strategies.

Name	LTR	LTR binder	POI binder	Connection mode	Targets	Target types	Cell line	LTR expression	*D*_max_ (%)	References
GFLD	Glut1	Small molecule	Antibody	Chemical conjugation	PD‐L1	Membrane	MDA‐MB‐231	6.4	65% (20 µg/mL)	[140]
GTAC	Glut	Small molecule	Antibody	Chemical conjugation	HER2	Membrane	SKBR3	4.2	85% (100 nM)	[141]
							SKOV3	5.0	80% (100 nM)	
							NCI‐N87	4.6	75% (100 nM)	
							BT474	5.7	85% (100 nM)	
					TNF‐α	Extracellular	SKBR3	4.2	/	
FRTAC	FR	Small molecule	Antibody	Chemical conjugation	EGFR	Membrane	Fadu	2.4	75% (100 nM)	[142]
					PD‐L1	Membrane	MDA‐MB‐231	5.1	75% (10 nM)	
							A549	0.3	60% (10 nM)	
					CD47	Membrane	MDA‐MB‐231	5.1	50% (100 nM)	
							A549	0.3	50% (100 nM)	
cli‐LYTAC	CD206	Small molecule	Small molecule	Chemical synthesis	Amyloid fibrils	Extracellular	BV2	/	/	[143]
GLP‐1–LYTAC	GLP‐1R	Peptide	Antibody	Chemical conjugation	EGFR	Membrane	HeLa	/	88% (100 nM)	[144]
							A549	/	63% (100 nM)	
					PD‐L1	Membrane	MDA‐MB‐231	/	82% (100 nM)	
GLTAC	GPC3	Peptide	Small molecule	Chemical synthesis	PD‐L1	Membrane	HepG2	3749.6	99.99% (10 µM)	[145]
					c‐Met	Membrane	HepG2	3749.6	71% (10 µM)	
							Caco2	207.0	87% (10 µM)	
					FGFR1	Membrane	HepG2	3749.6	85% (10 µM)	
							Caco2	207.0	99.96% (10 µM)	
DENTAC	SRs	Aptamer	Aptamer	Chemical conjugation	NCL	Membrane	A549	61.2	88% (50 nM)	[146]
			Antibody		EGFR	Membrane			72% (25 nM)	
TransMoDEs	Clathrin	Peptide	Small molecule	Chemical synthesis	Streptavidin	Extracellular	BEnd.3	/	/	[147]
Supra‐LYTAC	CAIX	Small molecule	Small molecule	Self‐assembly	PD‐L1	Membrane	HeLa	1.3	85% (100 µM)	[148]

TPD strategies that use cancer‐specific protein expression have great potential to improve treatment precision. However, a critical bottleneck is the limited availability of ligands selective for cancer cells. The glucose transporter GLUT1, overexpressed in diverse malignancies, offers dual advantages: it exploits the GLUT1‐mediated glucose uptake to fuel anaerobic glycolysis (the Warburg effect); and it harnesses GLUT1's natural endocytic recycling pathway, wherein the receptor continuously shuttles between membrane and early endosomes [149, 150]. This intrinsic recycling pathway positions GLUT1 as an ideal LTR for TPD [151]. In 2024, Geng et al. were the first to develop the GFLD technique by harnessing GLUT1 endocytosis activity to degrade membrane proteins [140]. Glycooligomer conjugates with variable monomer ratios were synthesized and linked with avelumab. The glycooligomer interacts with GLUT1 while avelumab binds with PD‐L1 to form a ternary complex. This efficiently degrades PD‐L1, thereby arresting immune evasion mechanisms in cancer cells. The Cai group also enhanced this concept with GTAC by conjugating multiple glucose molecules with an antibody against the POI with click chemistry [141]. They found that GTACs induced lysosomal degradation of HER2 and TNF‐α, suppressing proliferation in N87 and MDA‐MB‐231 cells. Furthermore, in N87 xenograft models, GTACs outperformed parental antibodies, achieving enhanced tumor regression with minimal off‐target toxicity. Therefore, the GTAC platform represents a novel degradation strategy that leverages GLUT1 for the tumor‐selective depletion of secreted and membrane POIs. These GLUT1‐driven TPD strategies offer several key advantages, including tumor‐selective degradation enabled by GLUT1's frequent overexpression in malignancies; broad adaptability to diverse targets such as immune checkpoints and growth factor receptors; and metabolic synergy, as this approach inherently targets the Warburg effect.

FR is overexpressed in several cancers but is largely absent from most healthy tissues, positioning it as an important biomarker and therapeutic target [152]. Beyond the classic role of FR in diagnostics and drug delivery, its natural endocytic recycling pathway has been strategically used for TPD [153]. Based on early work with folate‐conjugated PROTACs, Tang et al. made folate receptor‐targeting chimeras (FRTACs). This is a two‐part platform designed for tumor‐selective degradation of membrane proteins [142, 154]. The FRTAC design conjugates folate, which targets the FR, to an antibody specific for a membrane POI (like EGFR, PD‐L1). This creates a ternary complex that triggers clathrin‐mediated endocytosis, shuttling the entire complex to lysosomes for degradation of the oncogenic target. This mechanism successfully degraded EGFR and PD‐L1 in FR‐high cancer cells, and it suppressed growth and immune checkpoint signaling. Importantly, in multiple xenograft models, FRTACs worked better than conditional antibodies. They achieved over 50% tumor growth inhibition with no obvious body‐wide toxicity. FRTACs offer several advantages: they allow tumor‐selective degradation through FR targeting; they have plug‐and‐play adaptability because the platform works with any antibodies targeting POIs; and they have streamlined production, simplifying the development compared with custom synthesis needed for many PROTACs.

The accumulation of extracellular toxic Aβ plaques in the brain is a key feature of Alzheimer's disease. Antibody‐based treatments targeting Aβ have shown promise, but face problems. These include nonspecific immune activation through Fc receptors and limited access to binding sites on different Aβ aggregates [155]. These limitations underscore the need for alternative therapeutic strategies. LYTACs were first made for cancer therapy and have worked well in degrading secreted proteins (like ApoE4) and membrane targets (like PD‐L1 and EGFR). Based on this platform, Qu et al. adapted LYTAC technology for Alzheimer's disease. They made multifunctional polydopamine‐based LYTACs (KPLYs). These are named click chemistry‐enabled LYTACs (cli‐LYTACs), and they address key barriers in neurodegenerative treatment [143]. Unlike standard antibody‐dependent LYTACs, which have poor BBB permeability, cli‐LYTACs use a unique in situ synthesis mechanism. In Alzheimer's disease lesions, two precursors react through an Aβ‐Cu^2+^‐catalyzed click chemistry reaction. This is a bioorthogonal process that allows selective molecular assembly in biological environments, and forms activated cli‐LYTACs. These activated chimeras bridge Aβ aggregates to the lysosomal shuttling receptor CD206, which facilitates Aβ internalization and degradation in lysosomes. Beyond Aβ clearance, KPLYs improve treatment through three synergistic mechanisms. First, they upregulate CD206, which boosts receptor expression to improve Aβ uptake and degradation. Second, they have anti‐inflammatory effects that reduce chronic brain inflammation, which is a key driver of Alzheimer's disease progression. Third, they scavenge ROS, which removes ROS to reduce oxidative stress in brain tissues [156]. This activable platform circumvents BBB limitations and eliminates reliance on antibodies, offering a targeted, multifunctional approach for Alzheimer's disease.

Studies reported that the TfR, FR, and GLP‐1R can help deliver dyes, drugs, or nanomaterials into cells through receptor‐mediated uptake [157, 158, 159]. Of these receptors, GLP‐1R is a class B GPCR. It is widely expressed in many tissues and plays a key role in glucose homeostasis by binding its natural ligand, GLP‐1 [160, 161]. Beyond metabolic control, GLP‐1R has unique uptake properties. Upon ligand binding, the GLP‐1/GLP‐1R complex undergoes internalization. GLP‐1 goes to lysosomes while GLP‐1R recycles back to the cell membrane through early endosomes [162]. This distinctive trafficking behavior has inspired its repurposing for drug delivery, including antisense oligonucleotides and estrogen conjugates. Based on this, Ge et al. made a hypothesis that GLP‐1R can be exploited for lysosome‐TPD and thus developed the GLP‐1–LYTACs as a novel class of protein degraders [144]. GLP‐1–LYTACs were made by connecting GLP‐1 to antibodies using bioorthogonal click chemistry. This is a strong method that allows precise linking without disrupting molecular function. This modular design uses two key features: it ensures receptor‐mediated uptake through GLP‐1R's trafficking pathway, and it directs degradation to extracellular or membrane‐bound target proteins. As a proof‐of‐concept, GLP‐1–LYTACs degraded both extracellular proteins (like GFP and Neutravidin) and membrane proteins (like EGFR and PD‐L1) in a time‐ and concentration‐dependent way. The GLP‐1–LYTAC platform offers several advantages. It can quickly adapt to new targets through exchange of antibody domains, allowing customization for different target proteins. It has dual therapeutic use across disease areas, shown by targeting GLP‐1R for metabolic disorders and cancer immunotherapy. And it has better specificity, achieved by using the tissue‐specific expression pattern of receptors like GLP‐1R to reduce off‐target effects.

HS proteoglycans (HSPGs), especially GPC3, are cell‐surface receptors involved in the uptake of large molecular cargo [163]. GPC3 is a glycosylphosphatidylinositol‐anchored HSPG. It is overexpressed in over 70% of liver cancers and is linked to tumor growth, metastasis, and poor prognosis [164, 165]. Beyond its diagnostic use, GPC3's endocytic recycling pathway makes it an ideal LTR for cancer‐selective protein degradation. Sheng et al. made GLyPican‐3‐TArgeting Chimeras (GLTACs) and showed the mechanism using GPC3‐targeted photosensitizers [166]. This is a bifunctional degradation platform with three components: a GPC3‐targeting peptide; a target protein ligand that binds membrane proteins critical for tumor survival (like PD‐L1, c‐Met, or FGFR1); and a chemical linker that optimizes spatial flexibility to ensure ternary complex formation [145]. The formation of a ternary complex with GPC3, GLTAC, and target protein promotes uptake and then induce lysosomal degradation of membrane proteins. As proof‐of‐concept, GLTACs significantly removed various membrane proteins, including PD‐L1, c‐Met, and FGFR1, in HepG2 and Caco2 cells. Because GPC3 is specifically expressed in tumors, GLTACs can selectively degrade membrane proteins in cancer cells, offering a novel and powerful strategy for degrading tumor‐specific membrane proteins.

SRs are a diverse family of cell‐surface receptors. They are best known for binding and taking in modified LDL and other polyanionic ligands [167]. Beyond lipid metabolism, SRs facilitate the uptake of pathogens, apoptotic cells, and nucleic acid nanomaterials through the endosomal–lysosomal pathway [168]. SRs are a large family of cell‐surface receptors that were initially identified for their ability to bind and internalize modified LDL. They are now known to bind many ligands, including endogenous proteins and pathogens. DNA nanomaterials can specifically bind to SRs and are then taken in through the endosomal–lysosomal pathway. So SRs have been used for cargo delivery in DNA nanotechnology [169]. Importantly, SRs are highly expressed on cancer cells, and this helps deliver therapeutic agents based on nucleic acid nanodevices into cells [170]. These findings suggest the potential of using SRs as targeting receptors to mediate membrane protein degradation. To explore this potential, Li et al. made a dendronized DNA chimera (DENTAC), which includes a ligand targeting POIs and a DNA dendron targeting SRs [146]. As proof‐of‐concept, DENTACs achieved significant degradation of NCL (a ribonucleoprotein critical for tumor growth) and EGFR, demonstrating its ability to move the protein target into the lysosome for degradation. Also, in A549 xenograft models, NCL‐targeting DENTACs suppressed tumor growth, proving a therapeutic potential. Overall, the DENTACs platform offers several advantages: it is modular since ligands can be easily changed to induce degradation of different membrane proteins; it works with antibodies, peptides, and small molecules for broad use; it has tumor selectivity because of the overexpression of SRs in tumors; it has synthetic flexibility because the programmable self‐assembly of DNA dendrons allows precise control over valency and geometry. Above all, the DENTAC platform combines the benefits of nucleic acid nanotechnology and receptor biology to enable cancer‐selective protein degradation.

The BBB's tight junctions restrict passive diffusion of hydrophilic and large molecules. These molecules need transport proteins, receptor‐mediated transcytosis, or adsorptive‐mediated transcytosis to enter the brain [171]. To facilitate access to the central nervous system (CNS), peptides that can drive receptor‐mediated BBB transcytosis have been used [172]. Angiopep‐2 is a 19‐amino acid peptide from aprotinin's Kunitz domain. It targets the LDLR‐related protein 1 (LRP1) on BBB endothelial cells. Also, it exhibited a success in redirecting small molecules, antibodies, and NPs to brain both in vitro and in vivo [173]. Based on these findings, Spiegel et al. made transcytosis‐inducing MoDE proteins (TransMoDEs), which was based on their earlier work with MoDE proteins [147]. TransMoDEs are derived from Angiopep‐2, a peptide motif previously used as a covalent tag to facilitate receptor‐mediated transcytosis across the BBB. They can induce the endocytosis of proteins like streptavidin or HaloTag. In a bEnd.3 BBB model, TransMoDE‐mediated uptake of streptavidin happens through a clathrin‐mediated mechanism, resulting in both lysosomal localization and transcytosis of POI. Overall, TransMoDEs represent a great strategy to overcome BBB limitations, merging targeted drug delivery with extracellular protein degradation.

Supramolecular self‐assembly is driven by noncovalent interactions like hydrogen bonding and π–π stacking. It has revolutionized the design of dynamic, multifunctional nanomaterials [174]. Its ability to make multivalent structures with adjustable amounts has inspired new approaches in TPD, including supramolecular PROTACs [175, 176]. But achieving spatiotemporal control over extracellular chimeric structures is critical for precise degradation in biological environments. This remains a significant challenge. In 2025, Ryu et al. demonstrated that CAIX is a hypoxia‐inducible enzyme overexpressed in tumors. It mediates the uptake of extracellularly assembled nanofibers [177]. Leveraging this insight, they engineered CAIX‐targeting supramolecular nanofibrous LYTACs (Supra‐LYTACs). This platform combines three core components: first, a CAIX‐binding motif targets the tumor‐specific CAIX protein on cancer cell membranes. Second, a target protein‐interacting domain engages specific target proteins through tailored molecular interactions. Third, a self‐assembling peptide backbone is made of amphiphilic FFK (Phe–Phe–Lys) peptides that spontaneously form β‐sheet‐rich nanofibers under physiological conditions [148]. As proof‐of‐concept, they designed hetero‐functionalized self‐assemble amphiphilic FFK‐based peptides. These target the CAIX enzyme expressed on cancer cell membranes and interact with target proteins. They confirmed that Supra‐LYTAC can degrade both extracellular proteins (like streptavidin) and membrane proteins (like PD‐L1). Also, they showed in a 4T1 mouse breast cancer model that Supra‐LYTACs reduced tumor PD‐L1 levels by 50%, improving antitumor immunity. Supra‐LYTACs offer several advantages: they have tumor‐specific activation because self‐assembly is triggered mainly in CAIX‐rich tumor environments, so this minimizes off‐target effects; they have multivalent engagement because the resulting nanofibers improve binding strength through clustered presentation of both POI‐targeting and CAIX‐targeting motifs; and they have dynamic adaptability because the reversible nature of supramolecular structures allows for responsive degradation activity. By exploiting CAIX's dual role as a tumor marker and endocytosis mediator, this strategy achieves spatiotemporally controlled degradation, offering a versatile tool for precision oncology.

### 5.2 Degradation of Membrane Proteins by Recruitment of Intracellular Proteins

**FIGURE 3:**
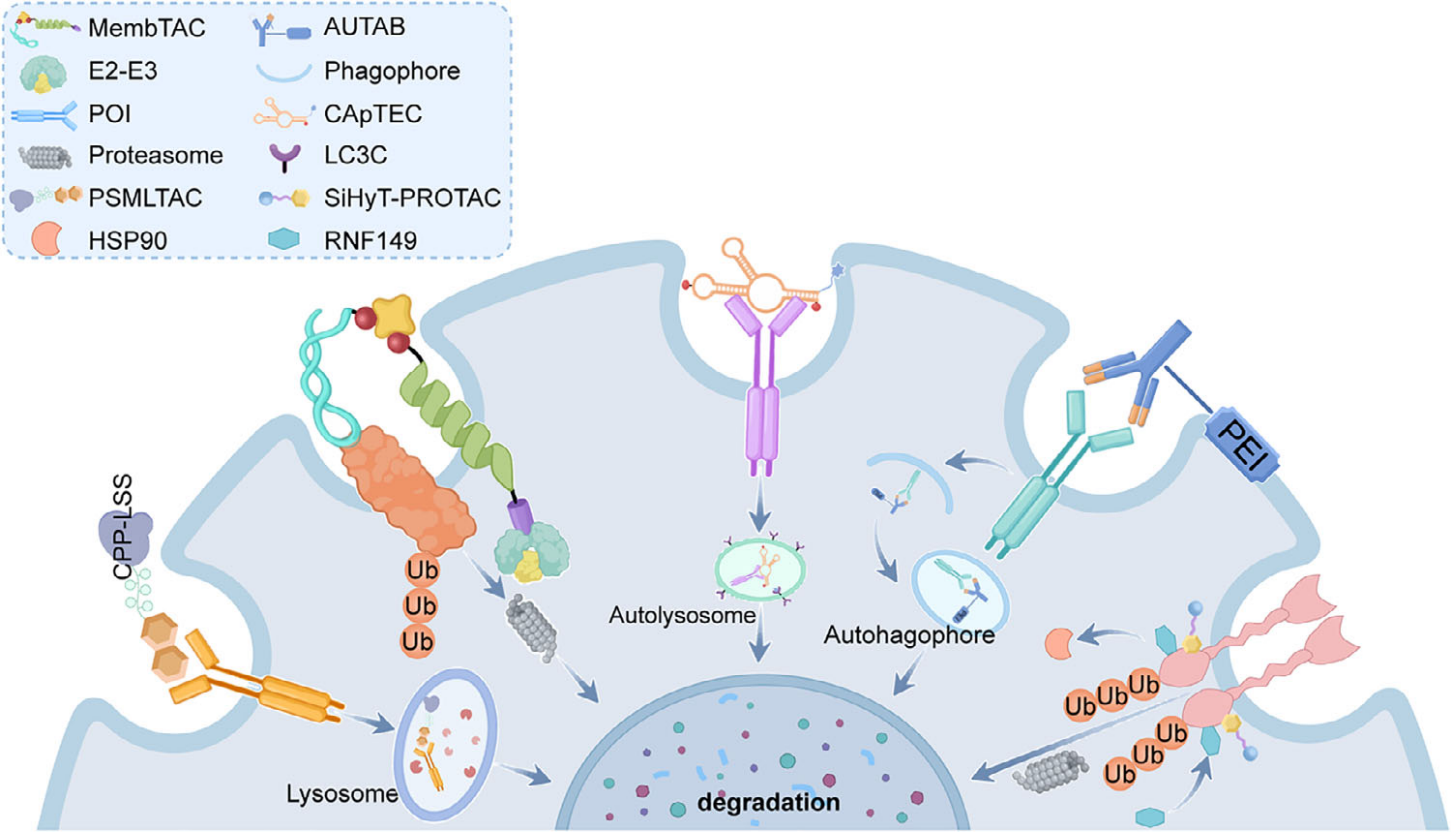
Schematic illustration of degraders that recruit intracellular proteins. The first approach utilizes LSS, which is a short peptide motif natively recognized by the lysosomal trafficking machinery. The second approach engineers molecules that simultaneously bind both LC3C and a POI, and POI can be moved into autophagosomes for lysosomal degradation. The third approach recruits E3 ligases like RNF149 and CRBN that will then tag POIs for degradation by the proteasome.

The TPD field has expanded beyond traditional LTR‐dependent approaches. It now includes new strategies using intracellular lysosomal sorting signals (LSS), ubiquitin–proteasome machinery, and autophagy pathways. These methods work with or bypass LTRs, and they broaden the scope of degradable targets, including extracellular secreted and membrane proteins. Below, we outline three key strategies and their applications (Figure 3). The first approach utilizes LSS, which is a short peptide motif recognized by the lysosomal trafficking machinery. Connecting LSS to target proteins reroutes them directly to lysosomes through interactions with adaptor proteins like AP‐1 or GGAs. This approach was effective for degrading intracellular proteins and displays significant promise for reducing disease‐causing proteins for a variety of disease conditions. A more classical approach within TPD research is the recruitment strategy for E3 ligases like RNF149 and CRBN that will then tag membrane proteins for degradation by the proteasome. Chimeric molecules, such as PROTACs, are designed to recruit E3 ligases and bring them into proximity with specific targets. This approach has successfully degraded oncogenic and neuropathogenic membrane proteins, and it highlights potential for treating cancer and neurodegenerative disorders. Finally, LC3C‐targeted degradation leverages the autophagy machinery. LC3C is a core autophagy protein embedded in the autophagosomal membrane. It recruits cargo through LC3‐interacting region motifs. By engineering molecules that simultaneously bind both LC3C and POIs, POIs can be moved into autophagosomes for lysosomal degradation. This strategy has been used to clear both cytosolic and membrane proteins, and it holds particular promise for treating diseases with pathogenic protein aggregation, such as Alzheimer's and Parkinson's disease. The table below summarizes intracellular proteins‐mediated TPD strategies, including the design details of key components, applicable target types, and maximum degradation efficiencies in each technology (Table 6).

**TABLE 6:** Summary of intracellular proteins mediated TPD strategies.

Name	Effector	Effector binder	POI binder	Connection mode	Targets	Target types	Cell line	*D*_max_ (%)	References
PSMLTAC	CPP‐LSS	Peptide	Small molecule	Chemical synthesis	PD‐L1	Membrane	A549	90% (2 µM)	[178]
SiHyT‐PROTAC	RNF149	Small molecule	Small molecule	Chemical synthesis	EGFR	Membrane	HCC‐827	98% (1 µM)	[179]
							PC‐9	80% (1 µM)	
MembTAC	CRBN	Small molecule	Aptamer	Biotin/streptavidin	EpCAM	Membrane	SW480	85% (20 nM)	[180]
					Met	Membrane	HeLa	87% (400 nM)	
CApTEC	LC3C	Small molecule	Aptamer	Chemical synthesis	TfR1	Membrane	HeLa	89% (250 nM)	[181]
					NCL			70% (75 nM)	
AUTAB	LC3C	PEI	Antibody	Chemical conjugation	PD‐L1	Membrane	MDA‐MB‐231	87% (25 nM)	[182]
							U87‐MG	90% (25 nM)	
							H292	84% (25 nM)	
							H1975	94% (25 nM)	
							A375	79% (25 nM)	
					EGFR	Membrane	HeLa	66% (25 nM)	
							A549	56% (25 nM)	
							Huh7	55% (25 nM)	
							PANC‐1	69% (25 nM)	
					CD73	Membrane	U87‐MG	62% (100 nM)	
							MDA‐MB‐231	52% (100 nM)	
							H1299	60% (100 nM)	
							PANC‐1	50% (100 nM)	
					Integrin α5	Membrane	H1299	38% (25 nM)	

#### 5.2.1 Degradation of Membrane Proteins by Recruitment of Intracellular Lysosome Sorting Sequence Recognition Proteins

LYTACs rely on cell‐surface LTRs to shuttle membrane proteins to lysosomes. However, low LTR expression in certain cancers limits degradation efficacy. So alternative strategies are needed to improve lysosomal delivery of target proteins. To avoid LTR dependency, SignalTACs use LSS, such as the NPGY motif, which are recognized by clathrin adaptor proteins (e.g., AP‐2) [183]. These chimeras fuse POI‐binding domains (like antibodies, peptides) with LSS peptides, enabling clathrin‐mediated endocytosis and lysosomal trafficking. The Cai and Chen et al. demonstrated LSS‐conjugated cell‐penetrating peptides (CPPs) degrade EGFR and PD‐L1 in low‐LTR cancer models [184, 185]. While most LYTACs employ antibodies or nanobodies, the Sheng et al. pioneered peptide‐mediated small molecule LYTACs (PSMLTACs), a fully synthetic platform combining: a POI Ligand targeting intracellular/membrane proteins, a LSS motif directing lysosomal sorting via clathrin recognition, and CPP enhancing cellular uptake (like TAT peptide) [178]. PSMLTACs degraded PDEδ (intracellular) and PD‐L1 (membrane) with >70% efficiency, outperforming PROTACs in direct comparisons. Additionally, the target scope of PSMLTACs was expanded to intracellular proteins nicotinamide phosphoribosyltransferase and BTK (Bruton's tyrosine kinase), demonstrating its versatility. Notably, this work aims to validate the feasibility of the PSMLTAC strategy, and further optimization of the linker may yield more potent degraders. Above all, this proof‐of‐concept study highlights the advantages of the PSMLTAC strategy and provides a broad applicability and efficient degradation tag for the development of protein degraders. Nevertheless, for PSMLTAC degraders to become therapeutically effective, they need to overcome potential limitations related to metabolic stability and oral bioavailability. Overall, PSMLTACs represent a transformative advance in TPD, merging small‐molecule precision with peptide‐enhanced lysosomal trafficking. By decoupling from LTRs and leveraging clathrin‐mediated pathways, this platform addresses critical gaps in degrading intracellular targets in low‐LTR cancers.

#### 5.2.2 Degradation of Membrane Proteins by Recruitment of the Intracellular E3 Ligase

Since the Crews et al. first confirmed the proof‐of‐concept for TPD using PROTACs, over 20 PROTACs are now in clinical trials. The vast majority of these PROTACs employ CRBN and VHL as E3 ligases, while additional E3 ligases are being explored to expand the application of PROTACs across different cell lines and tissues [186, 187, 188, 189]. The hydrophobic tag (HyT) strategy, which mimics exposed hydrophobic amino acids on protein surfaces and marks them for degradation as misfolded proteins. Building on this, the Yang et al. replaced carbon‐based HyTs with silicon‐based analogs (SiHyT), enhancing hydrophobicity and stability [179]. They designed a series of EGFR PROTACs by linking gefitinib with SiHyT through various chemical linkers and evaluated their efficacy. Among these compounds, degrader 7, which links gefitinib to a TBDPS silyl ether, achieved the most efficient EGFR degradation both in vitro and in vivo. Notably, it demonstrated 8.67% oral bioavailability, a notable advancement for PROTAC uptake. Also, the SiHyT strategy has been successfully used to design potent PD‐L1 and BTK degraders. It combines silicon chemistry's unique properties with innovative E3 ligase recruitment. By enabling oral bioavailability and expanding the range of degradable targets, this approach broadens the therapeutic potential of TPD.

PROTACs have been widely developed in both academic and industrial settings [176, 190]. But PROTACs face limitations in degrading membrane proteins because they rely on cytoplasmic E3 ligases. LYTACs can degrade membrane and extracellular proteins, but they face challenges such as large molecular weight, limited permeability, and more off‐target effects compared with PROTACs. To address the need for membrane protein degradation using adaptable PROTAC technologies, Zhu et al. developed membrane‐bound E3 ubiquitin ligase‐targeting chimeras (MembTACs) [180]. These MembTACs use intracellular E3 ubiquitin ligases to degrade membrane proteins. A key part of their system is the pH low insertion peptide (pHLIP). This can insert into the cell membrane of tumor tissue because of the low extracellular pH (6–6.8), but not at physiological pH (7.2–7.4). In the MembTAC system, the C‐terminus of pHLIP is designed to insert into the cell membrane at the low pH of tumor tissues, while the N‐terminus of pHLIP is connected to biotinylated aptamers targeting membrane proteins via a streptavidin linkage. When the pHLIP inserts into membranes in acidic tumor areas, it positions the aptamer–E3 ligase complex for target engagement. As proof‐of‐concept, the Zhu et al. designed two MembTACs: SYL3C_1_‐(pHLIP‐P)_3_, using B‐SYL3C as an aptamer targeting EpCAM, and Mapt_1_‐(pHLIP‐P)_3_, using Mapt as an aptamer targeting Met. Both MembTACs degraded the EpCAM and Met, respectively, demonstrating the potential of MembTACs as a potent and flexible platform for degrading membrane proteins. MembTACs offer several advantages: They have tumor specificity because the pHLIP peptide ensures activation mainly within acidic tumor environments; They are modular because streptavidin–biotin linkages allow quick adaptation to new targets through simple aptamer exchange; They have a compact molecular size of about ∼10–20 kDa, which greatly improves tissue penetration compared with the ∼200 kDa typical of classic LYTACs; and they use intracellular E3 ligases, which use the potent cytoplasmic degradation machinery (like CRBN) and avoid the reliance on lysosomal receptors required by LYTAC platforms. Overall, MembTACs represent a breakthrough in membrane protein degradation, merging pH‐sensitive targeting with PROTAC‐like E3 ligase recruitment.

#### 5.2.3 Degradation of Membrane Proteins by Recruitment of the Intracellular LC3C

At the early stage of classic autophagy induction, molecules like mTOR inhibitors initiate a signaling chain. This leads to the new formation of a double‐membraned phagophore that nonselectively engulfs cytoplasmic content. It matures an autophagosome and fuses with a lysosome. There are selective forms, but a classic inducer mainly boosts this bulk process [191]. An LC3C‐mediated TPD degrader works as a precision tool at the downstream execution point. In the design of degrader, one end binds the POI, and the other end binds LC3C proteins already anchored to the membrane of a forming autophagosome. It physically crosslinks the POI directly to the degradation machinery, ensuring its selective inclusion. This “hijacks” the final stage, forcing the autophagosome to engulf a specific cargo without the need for cell‐wide induction, resulting in enhanced targeted degradation [192]. Similar for TPD, aptamer‐based chimeras, such as LYTACs, have been engineered to degrade membrane proteins by bridging LTRs or E3 ubiquitin ligases to POIs [193]. However, these approaches face limitations due to variable expression of LTRs/E3 ligases across tissues, structural instability of aptamers, and off‐target effects. To address these problems, Zhang et al. introduced covalent aptamer‐based autophagosome‐tethering chimeras (CApTECs). This is a new platform using covalent chemistry and autophagy hijacking [181]. CApTECs combine two key innovations: covalent “warhead” (ASF) and LC3 ligand connection. In their design, ASF selectively cross‐links aptamers to lysine, tyrosine, or histidine residues on target proteins, improving binding stability and specificity [85]. By tethering the aptamer to LC3, CApTECs hijack the autophagosome–lysosome pathway, avoiding traditional LTR/E3 ligase dependencies. So CApTECs significantly improved and prolonged the degradation of TfR1 and nucleolin, compared with the noncovalent ApTEC design. Also, Zhang et al. showed the synergistic tumor therapy of CApTEC with 5‐fluorouracil in a mouse model, providing an effective strategy for membrane protein degradation and precise therapeutic applications via covalent chemistry. CApTECs address key limitations of aptamer‐based TPD by combining covalent targeting with autophagy hijacking, enabling precise degradation of membrane proteins.

In the same way, Li et al. developed autophagy‐inducing antibodies (AUTABs), using polyethylenimine (PEI), a polymer known for gene delivery and autophagy induction [182, 194]. By conjugating PEI to therapeutic antibodies, they made AUTABs to degrade membrane proteins via autophagy, offering a new approach different from proteasome‐dependent methods like PROTACs or lysosome‐targeting LYTACs. As proof‐of‐concept, they made an AUTAB molecule called ATZ–AUTAB by connecting PEI to ATZ. They found that ATZ–AUTAB nearly removed PD‐L1 within 24 h and effectively degraded PD‐L1 in MDA‐MB‐231 xenografts, showing its potential as a promising PD‐L1 degrader. To expand the use of AUTABs to other membrane proteins, Li et al. made CTX–AUTAB and Ole–AUTAB using the same chemical method to study the degradation of EGFR and CD73, respectively. Similar to ATZ–AUTAB, both CTX–AUTAB and Ole–AUTAB significantly removed their respective target proteins in various cell lines. These results strongly showed the broad applicability of the AUTAB platform for targeting different types of membrane proteins. Also, to create nano‐AUTABs, Li et al. explored the feasibility of connecting PEI to a target receptor using a general secondary antibody, a commercially available secondary nanobody to mouse IgG. As expected, nano‐AUTABs could degrade multiple targets at the same time, as shown by the concurrent degradation of PD‐L1 and EGFR in MDA‐MB‐231 cells.

### 5.3 Degradation of Extracellular and Membrane Proteins Independent of Specific Surface Receptors and Intracellular Proteins

**FIGURE 4:**
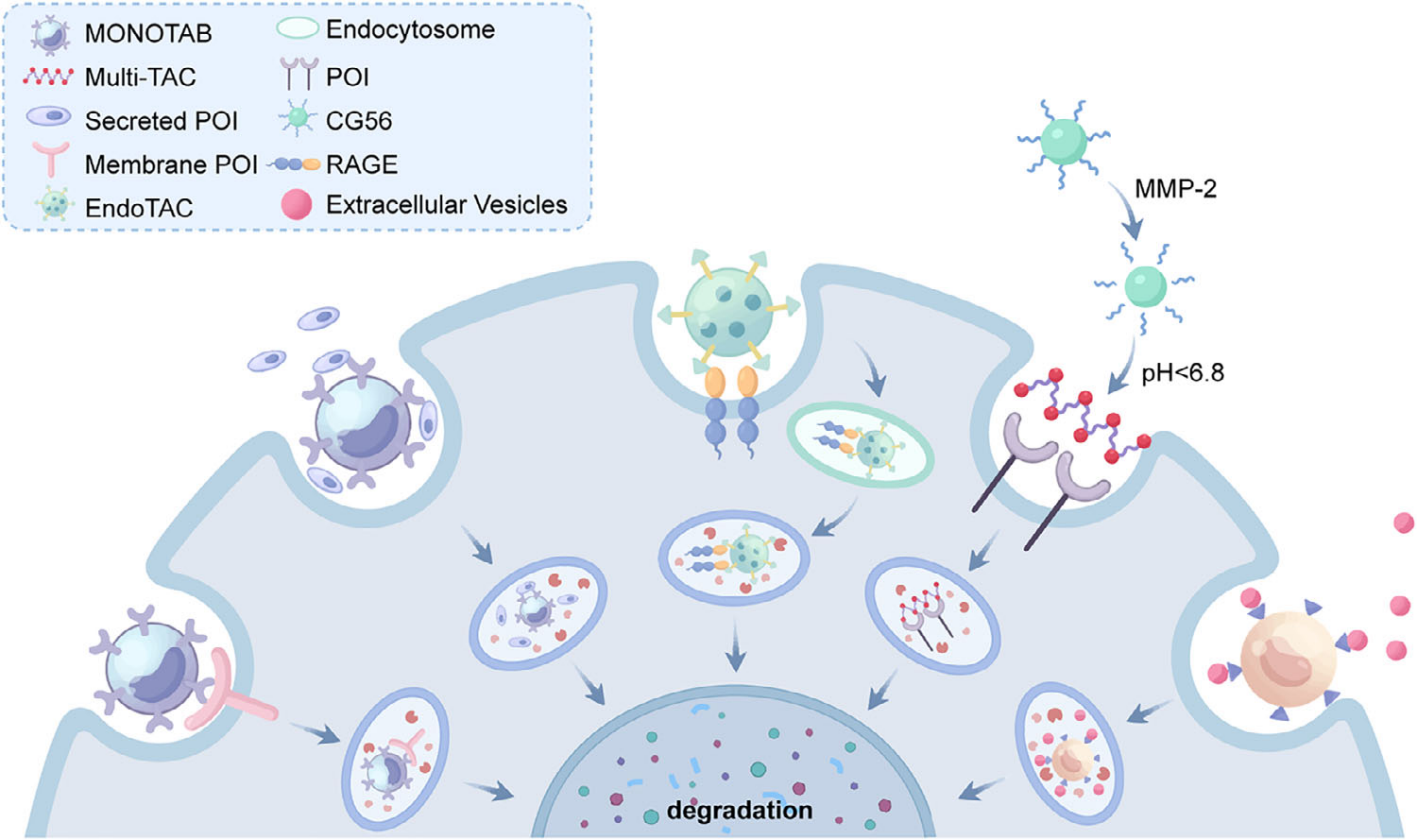
Schematic illustration of degraders that are independent on LTRs or intracellular proteins. NPs’ natural endosomal escape properties or engineered pH‐responsive coatings that promote delivery to lysosomes, leading to the degradation of extracellular proteins, membrane proteins, and EV.

LYTACs use cell‐surface LTRs to internalize and degrade extracellular or membrane‐bound proteins. But low LTR expression in some cancers (like glioblastoma and pancreatic cancer) limits their effectiveness. This problem has increased interest in nanomaterials as alternative platforms for lysosomal degradation, bypassing the need for LTRs entirely. NPs use natural cellular uptake pathways, enabling efficient entry and lysosomal trafficking independent of LTR availability (Figure 4) [195, 196]. The tunable design of NPs enables key functionalities for TPD: this is achieved through enhanced endocytosis, as NPs are passively internalized via clathrin‐mediated pathways, overcoming any dependency on specific LTRs; efficient lysosomal movement, driven by the NPs’ natural endosomal escape properties or engineered pH‐responsive coatings that promote delivery to lysosomes; and versatile functionalization, where surface modifications with antibodies or aptamers enable precise targeting of disease‐relevant proteins. The table below summarizes NP‐mediated TPD strategies, including the design details of key components, target types, and maximum degradation efficiencies in each technology (Table 7).

**TABLE 7:** Summary of NPs‐mediated TPD strategies.

Name	Carrier	POI binder	Connection mode	Targets	Target types	Cell line	*D*_max_ (%)	References
MONOTAB	NPs	Antibody	Self‐assembly	PD‐L1	Membrane	B16F10	95% (3.3 nM)	[197]
						Hepa1‐6	56% (3.3 nM)	
				MMP2	Extracellular	B16F10	60% (12 nM)	
						CT26	66% (12 nM)	
				EVs	Extracellular	B16F10	/	
EndoTAC	NPs	Antibody	Self‐assembly	RAGE	Extracellular	BEnd.3 cells	/	[198]
SM‐CMAD	NPs	Small molecule	Self‐assembly	AR	Membrane	VCAP	90% (100 µM)	[199]
						LNCAP	90% (100 µM)	
Multi‐TACs	NPs	Small molecule	Self‐assembly	PD‐L1	Membrane	B16F10	50% (10 µM)	[200]

The successful application of nanomaterials in delivering cargo to lysosomes has inspired the development of innovative TPD platforms. One such platform, developed by Shao et al., is the modified NPs with targeting binders (MONOTAB) [197]. This system utilizes the unique properties of NPs to reroute secreted or membrane proteins into lysosomes for degradation. In the preparation process of MONOTAB, the NPs are first connected with streptavidin, and then are assembled with biotinylated anti‐IgG antibodies. This platform provides a flexible and universal strategy with different antibodies to degrade target proteins, such as PD‐L1 and MMP2. By providing a flexible and universal strategy for degrading target proteins, MONOTAB can be used to develop new therapies for various diseases, including cancer and neurodegenerative disorders. Also, the platform's ability to degrade nonprotein targets like extracellular vesicles (EVs) opens up new paths for research into intercellular communication and disease mechanisms.

LYTAC technology has appeared as an effective strategy for protein degradation, especially for extracellular and membrane proteins. But the degradation of membrane proteins is often limited because of low lysosome‐sorting effectiveness. To address this challenge, Gao et al. developed a novel nano‐chimera called EndoTAC. It uses a polyvalent receptor binding mode to enhance the degradation of receptor for advanced glycation end products (RAGE). RAGE is a well‐known disease marker and drives the start and progression of Alzheimer's disease [198, 201]. In their design, the surface of the NP is changed with ligands that specifically target RAGE. So, this ensures that the EndoTAC gets delivered to the cells where there is a high expression of RAGE receptors. Also, the EndoTAC operates through polyvalent interactions. These interactions exhibit a high level of affinity and specificity, much like antigen–antibody interactions [202]. By using multiple ligands, EndoTAC can bind more strongly to RAGE. So this increases the efficiency of receptor uptake and degradation. The combination of LYTAC‐induced TPD and effective drug delivery systems like EndoTAC offers a promising strategy for Alzheimer's disease therapy. By degrading RAGE, EndoTAC can potentially reduce the buildup of amyloid‐beta plaques and other toxic proteins. Also, delivery systems have the potential to enhance the efficacy of treatment used in neurodegenerative disorders. EndoTAC offers clear advantages over traditional LYTACs. These include improved lysosomal sorting through a NP design that enhances delivery efficiency and overcomes the low sorting effectiveness of conventional methods.

Chaperone‐mediated autophagy (CMA) is a selective lysosomal degradation pathway. It targets cytosolic proteins containing a KFERQ‐like pentapeptide motif. This motif is recognized by the chaperone protein HSC70, which directs substrate proteins to lysosomes for degradation. CMA plays critical roles in maintaining protein balance. It has been involved in degrading pathogenic proteins such as α‐synuclein (linked to Parkinson's disease), PD‐L1, and mutant huntingtin (associated with Huntington's disease) [203, 204, 205, 206, 207]. CMA chimeras are engineered molecules to selectively degrade target proteins through the CMA pathway. These chimeras typically have three essential parts: a cell‐penetrating domain that helps intracellular delivery; a protein‐binding domain that binds specifically to POI; and a KFERQ‐like motif that recruits HSC70 to route the target protein to lysosomes. But the development of CMA chimeras has been slowed by the complexity of rational design and linker optimization between the target protein ligands and effector molecules. Thus, this challenge has greatly limited the practical application and scalability of CMA chimeras [208]. Yin and Li et al. addressed these challenges by developing split‐and‐mix CMA‐based degraders (SM‐CMADs). This was inspired by their earlier work on split‐and‐mix PROTACs (SM‐PROTACs) [199, 209]. In their system, the tetrapeptide consists of diphenylglycine (δδ) forming spherical nanometric assemblies and two arginines (RR) for improved membrane penetration. This design greatly improves the chimera's ability to enter cells and reach its target. SM‐CMADs self‐assemble into functional nanostructures that efficiently enter cells, bind to target proteins, and recruit HSC70 through the KFERQ motif, triggering lysosomal degradation. This platform successfully degraded several membrane proteins, such as estrogen receptor α and androgen receptor through a lysosome‐dependent mechanism, providing an efficient strategy for precise cancer therapy.

LTR technology greatly expands the range of targetable proteins. This technology leverages ligands to direct proteins to lysosomes for degradation, offering a versatile strategy for treating various diseases, including cancer. But the clinical use of LTR‐based TPD technology is hindered by the lack of tumor‐specific expression of LTRs, limiting its therapeutic effectiveness and specificity [210]. To address this challenge, Xu et al. developed polymer‐based multivalent targeting chimeras (multi‐TACs) to achieve tumor‐selective membrane protein degradation [200]. The degradation of PD‐L1 was used as a model to show the proof‐of‐concept. Multi‐TACs are made by copolymerization of a hydrophilic methexyl PEG segment, an acid‐ionizable tertiary amine, and a small molecule PD‐L1 ligand BMS‐1. As designed, under neutral pH conditions of normal cells, multi‐TACs self‐assemble into micellar NPs with the PD‐L1 ligand enclosed within the hydrophobic core. This setup minimizes toxicity because of less PD‐L1 degradation. But in the acidic environment of cancer cells (pH 6.5–6.8), the tertiary amine groups in multi‐TACs undergo protonation, causing the NPs to dissociate and expose the PD‐L1 ligand. This activated state enables multi‐TACs to bind to the extracellular domain of PD‐L1, redirecting it for uptake through adsorption‐mediated endocytosis and subsequent lysosomal degradation. The optimized multi‐TACs, such as GG56, include an MMP‐2‐sensitive PEG segment and a pH‐responsive module. These modifications enhance PD‐L1 degradation effectiveness and antitumor performance both in vitro and in vivo. Notably, GG56 NPs efficiently degrade PD‐L1 without relying on membrane receptors or LTRs, demonstrating superior tumor selectivity and minimal off‐tumor effects. Further studies showed that GG56 NPs mediate PD‐L1 degradation through the lysosomal pathway, not through the proteasome or autophagy pathways. This lysosomal targeting ensures efficient and specific PD‐L1 degradation, improving the therapeutic effectiveness of multi‐TACs. Comparatively, the multi‐TACs platform offers several advantages over existing inhibitors and degraders of PD‐L1: it confers superior tumor selectivity owing to that its activation is specifically induced by the acidic pH and enzymatic conditions in the tumor environment; the platform further enhances the uptake of POI for degradation because GG56 NPs stick to the tumor cell membrane via electrostatic interactions; multi‐TACs exert versatile drug delivery capability, acting as powerful nano‐vectors for combined cancer immunotherapy. Such a multifunctional approach increases the overall therapeutic effectiveness.

All in all, the targeted degradation of extracellular and membrane proteins has redefined therapeutic goals, prioritizing degradation over inhibition in cancer therapy. By moving beyond simple inhibition to the degradation of key oncogenic drivers, this emerging modality expands the therapeutic arsenal. It offers the potential for more durable responses and overcomes resistance mechanisms, positioning itself as a next‐generation strategy in precision oncology. The table below summarizes the selected TPD strategies that have been successfully validated in preclinical models, aiming to provide a foundational primer for the rational design of next‐generation degraders (Table 8).

**TABLE 8:** Summary of selected TPD strategies including preclinical animal experiments.

Name	Effector	Cell/tissue selectivity	Pathway mechanism	Preclinical mouse model	References
TfR–LYTAC	TfR	Multiple types	Endocytosis–lysosomal	B16F10	[74]
				CT26	
TransTAC	TfR	Multiple types	Endocytosis–lysosomal	PC9Del19/T790M/C797S	[75]
Pep‐TAC	TfR	Multiple types	Endocytosis–lysosomal	MC38	[77]
IFLD	Integrin	Multiple types	Endocytosis–lysosomal	B16F10	[116]
aPDL1–RGD NPs	Integrin	Multiple types	Endocytosis–lysosomal	B16F10	[117]
ITGBACs	Integrin	Multiple types	Endocytosis–lysosomal	DU‐145	[119]
iLYTACs	IGF2R	Ubiquitous	Endocytosis–lysosomal	HeLa	[131]
IGF‐EndoTag	IGF2R	Ubiquitous	Endocytosis–lysosomal	A20	[132]
GTAC	Glut	Multiple types	Endocytosis–lysosomal	NCI‐N87	[141]
FRTAC	FR	Multiple types	Endocytosis–lysosomal	B16F10	[142]
				CT26	
				MOC1	
DENTAC	SRs	Multiple types	Endocytosis–lysosomal	A549	[146]
Supra‐LYTAC	CAIX	Multiple types	Endocytosis–lysosomal	4T1	[148]
CApTEC	LC3C	Ubiquitous	Autophagy–lysosomal	HeLa	[181]
AUTAB	LC3C	Ubiquitous	Autophagy–lysosomal	MC38	[182]
				MDA‐MB‐231	
MONOTAB	NPs	Ubiquitous	Endocytosis–lysosomal	B16F10	[197]
EndoTAC	NPs	Ubiquitous	Endocytosis–lysosomal	FAD^4T^	[198]
Multi‐TACs	NPs	Ubiquitous	Endocytosis–lysosomal	B16F10	[200]
				4T1	

## 6 Conclusion and Future Perspectives

In conclusion, the TPD field has successfully expanded its reach beyond the intracellular targets, launching a new chapter in therapeutic discovery aimed at extracellular and membrane proteins. This review has brought together the rapid development of new strategies, including LTR‐recruiting degraders, intracellular proteins‐engaging molecules, and receptor‐independent NP platforms. All of them address the critical limitation of traditional PROTACs. By using lysosomal trafficking or autophagy pathways, these technologies enable the complete removal of harmful proteins. This offers a potent solution to the challenges of drug resistance and the “undruggable” proteins that trouble conventional inhibition‐based therapies. The strong preclinical success across various animal models underscores the transformative potential of this approach to redefine the treatment of cancer, neurodegenerative disorders, and autoimmune diseases.

The next wave of innovation will focus on improving the precision and practicality of these degraders. Key prospects include the development of hybrid molecules, such as degrader–antibody conjugates or AOCs. These aim to combine the superior affinity and pharmacokinetics of antibodies with the catalytic efficiency of degradation modalities. Also, the use of tissue‐ or cell‐type‐specific LTRs and E3 ligases, guided by rich genomic and proteomic databases (like GTEx and TCGA), promises to minimize off‐target effects and bring in an era of truly precise therapeutic interventions. Moreover, overcoming delivery challenges, particularly for targets within the CNS, through advanced vector systems or BBB‐penetrating methods will be crucial for treating neurological diseases.

The application of these technologies will expand beyond oncology. The ability to clear harmful extracellular aggregates like amyloid‐β or α‐synuclein positions TPD as a promising strategy for neurodegenerative diseases. Also, elimination of inflammatory cytokines or immune checkpoints could revolutionize the management of autoimmune conditions. As the first wave of extracellular protein degraders moves toward clinical trials, the coming years will be critical for turning these scientific concepts into real medicines. The continued combination of chemical biology, structural insights, and engineering will ultimately unlock the full potential of TPD, paving the way for a new generation of therapies that remove the root causes of human diseases.

## Author Contributions

Mengqing Zhao, WenhaoYin, Jianjian Han, Huimin Wang, and Wuxiang Mao conducted the literature review and wrote the manuscript. Zheng Liu, Lilong Liu, and Wuxiang Mao proposed the topic of the review and supervised the manuscript preparation. All authors have read and approved the final manuscript.

## Funding

This study was supported by the National Key Research and Development Program of China (2025YFA0922400), National Natural Science Foundation of China (82303623), Noncommunicable Chronic Diseases‐National Science and Technology Major Project (2024ZD0525700), Science and Technology innovation Talent Plan of Hubei Province (2023DJC136), and Natural Science Foundation of Hubei Province (2025AFB825).

## Ethics Statement

The authors have nothing to report.

## Conflicts of Interest

The authors declare no conflicts of interest.

## Data Availability

All data are freely available from the corresponding author upon request.
